# Supplementary figures and images for: Histopathology of Thecaphora frezzii Colonization: A Detailed Analysis of Its Journey Through Peanut (Arachis hypogaea L.) Tissues
Source: Plants (Basel). 2025 Apr 1;14(7):1083. doi: 10.3390/plants14071083 (PMC11991206; doi:10.3390/plants14071083)

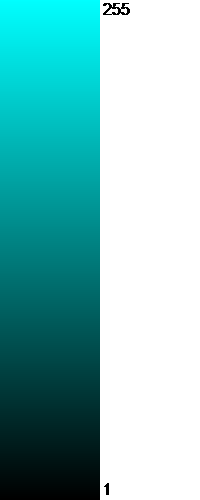

Supplement: Supplementary file 1 [file plants-14-01083-s001.zip › Appendix_A-CLSM-set-images_Fig8/MetaData/Florencia-calcofluor-241024_Series009_ch0_LUT_Cyan.png]

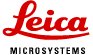

Supplement: Supplementary file 1 [file plants-14-01083-s001.zip › Appendix_A-CLSM-set-images_Fig8/MetaData/LeicaLogo.jpg]

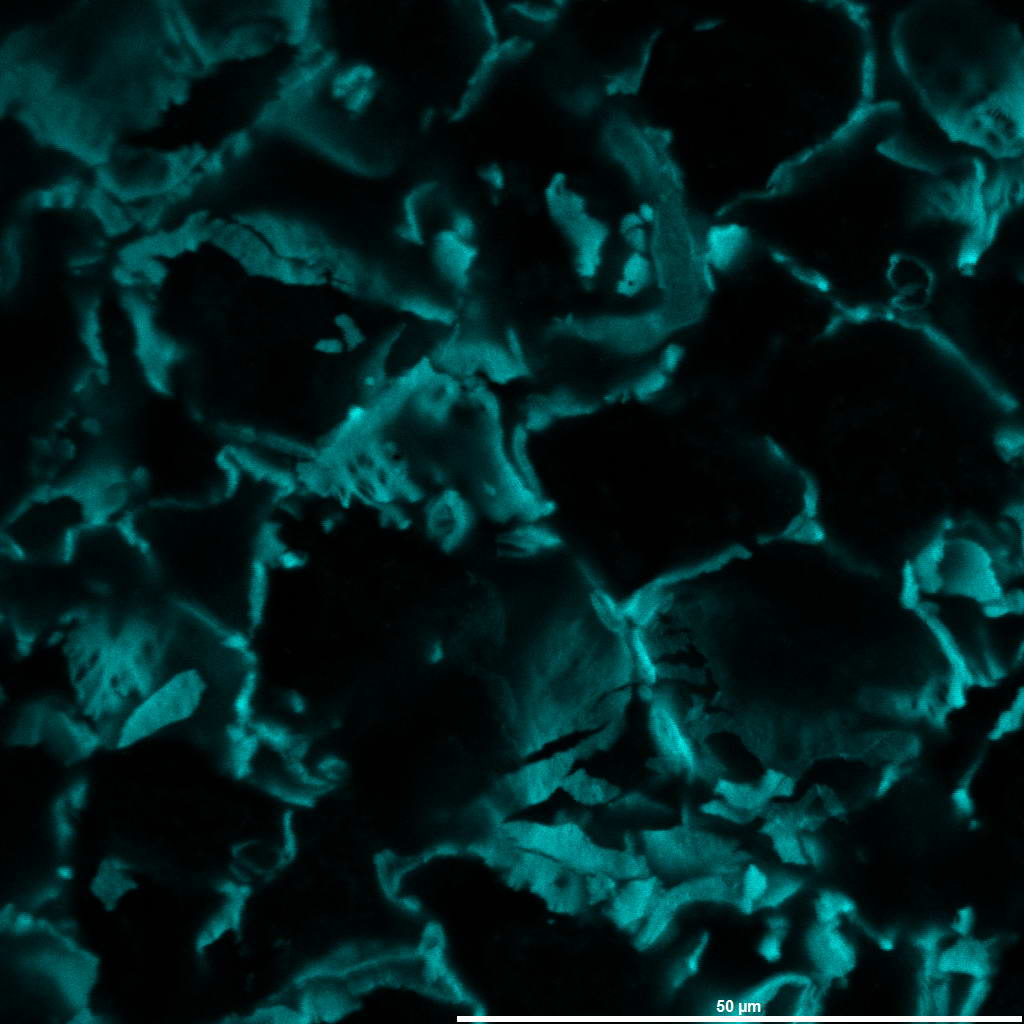

Supplement: Supplementary file 1 [file plants-14-01083-s001.zip › Appendix_A-CLSM-set-images_Fig8/Tfrezzi_peanut_CalcW_Ser09_z1.jpg]

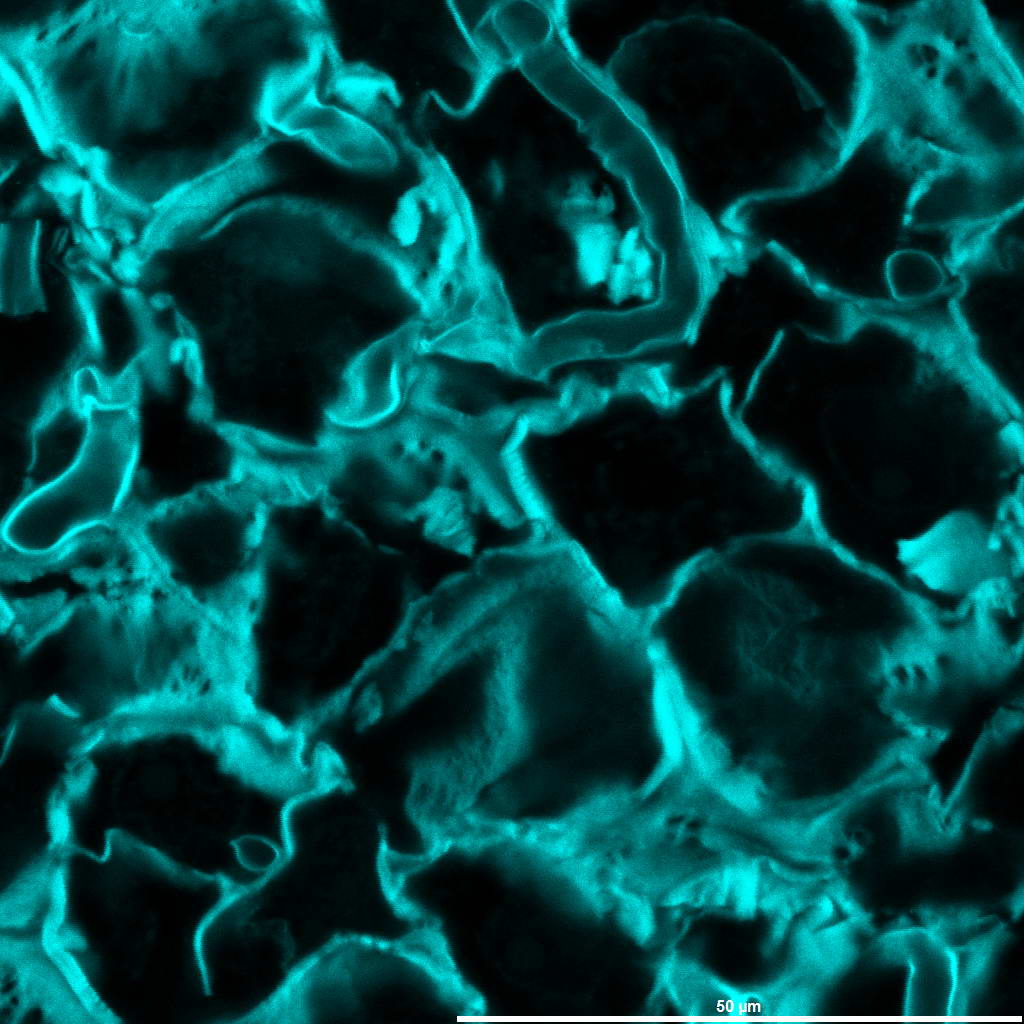

Supplement: Supplementary file 1 [file plants-14-01083-s001.zip › Appendix_A-CLSM-set-images_Fig8/Tfrezzi_peanut_CalcW_Ser09_z10.jpg]

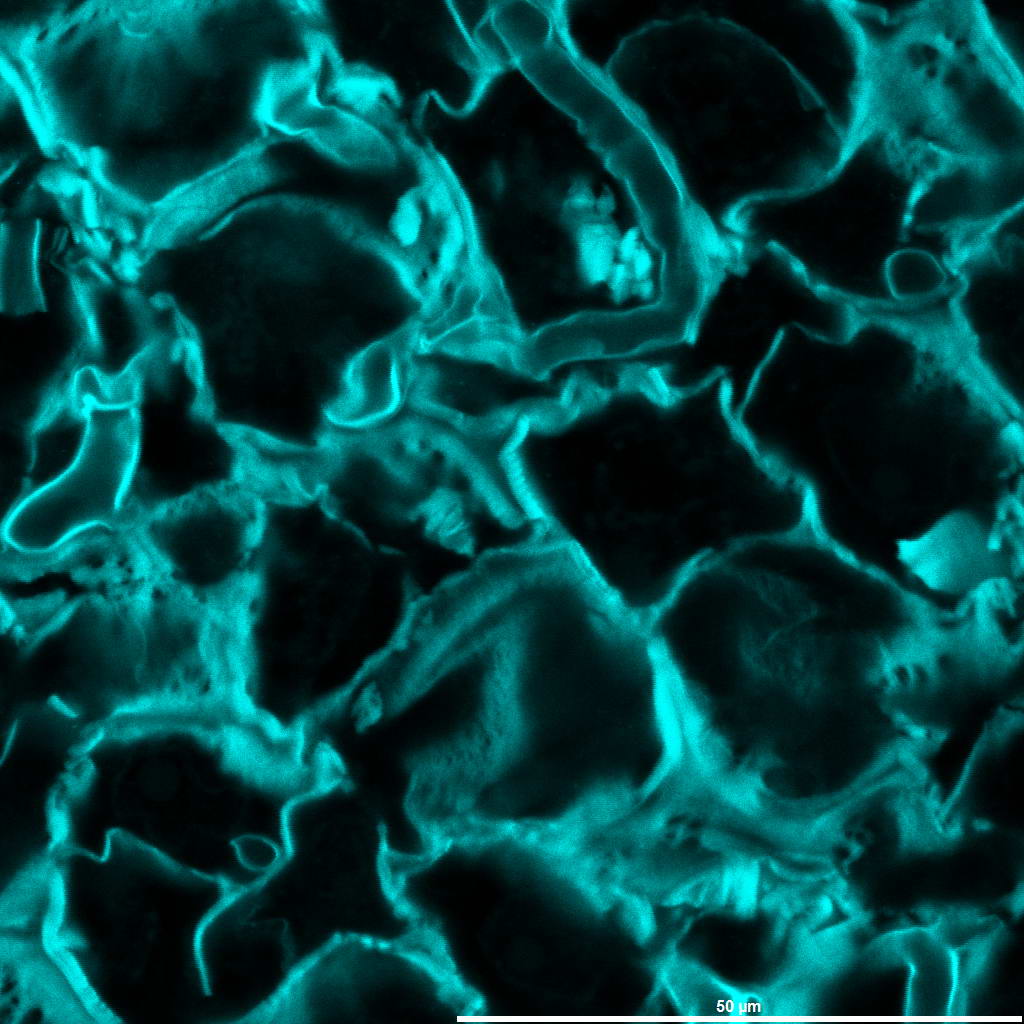

Supplement: Supplementary file 1 [file plants-14-01083-s001.zip › Appendix_A-CLSM-set-images_Fig8/Tfrezzi_peanut_CalcW_Ser09_z11.jpg]

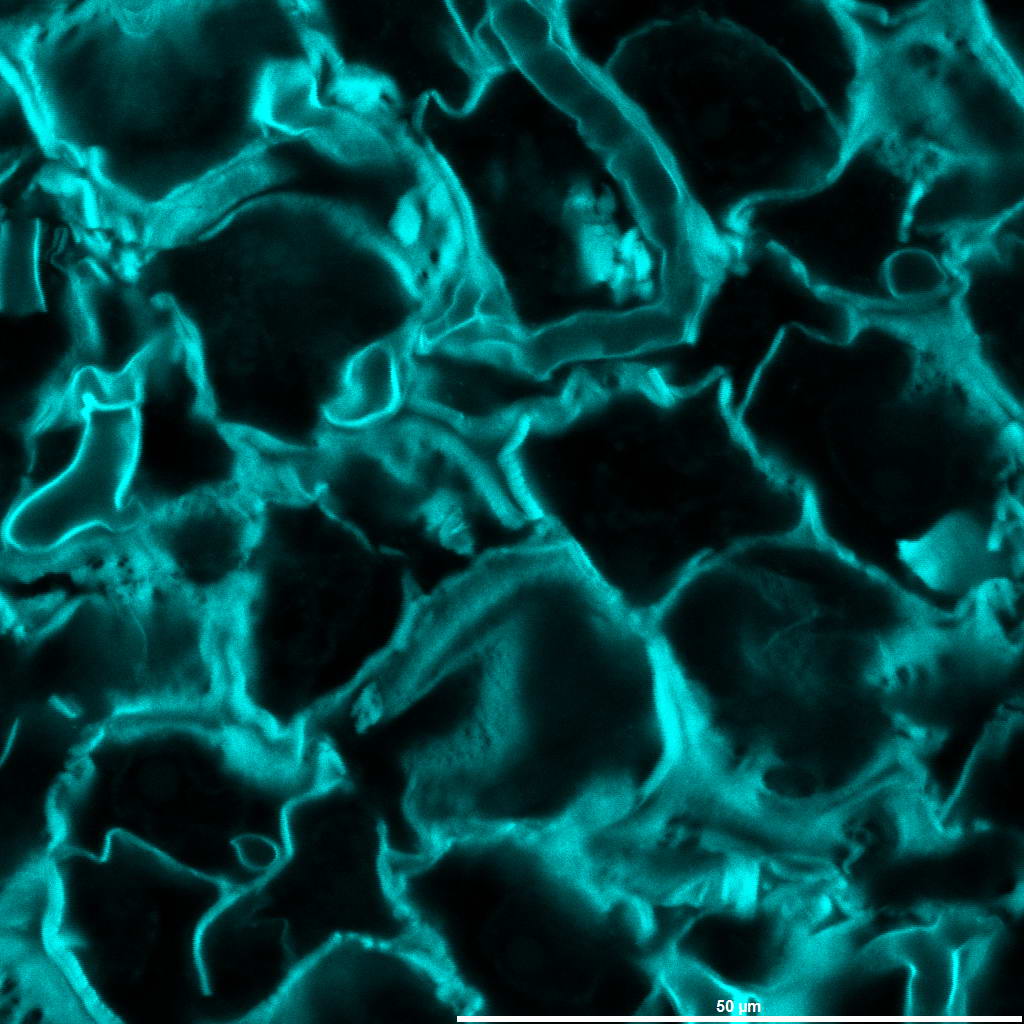

Supplement: Supplementary file 1 [file plants-14-01083-s001.zip › Appendix_A-CLSM-set-images_Fig8/Tfrezzi_peanut_CalcW_Ser09_z12.jpg]

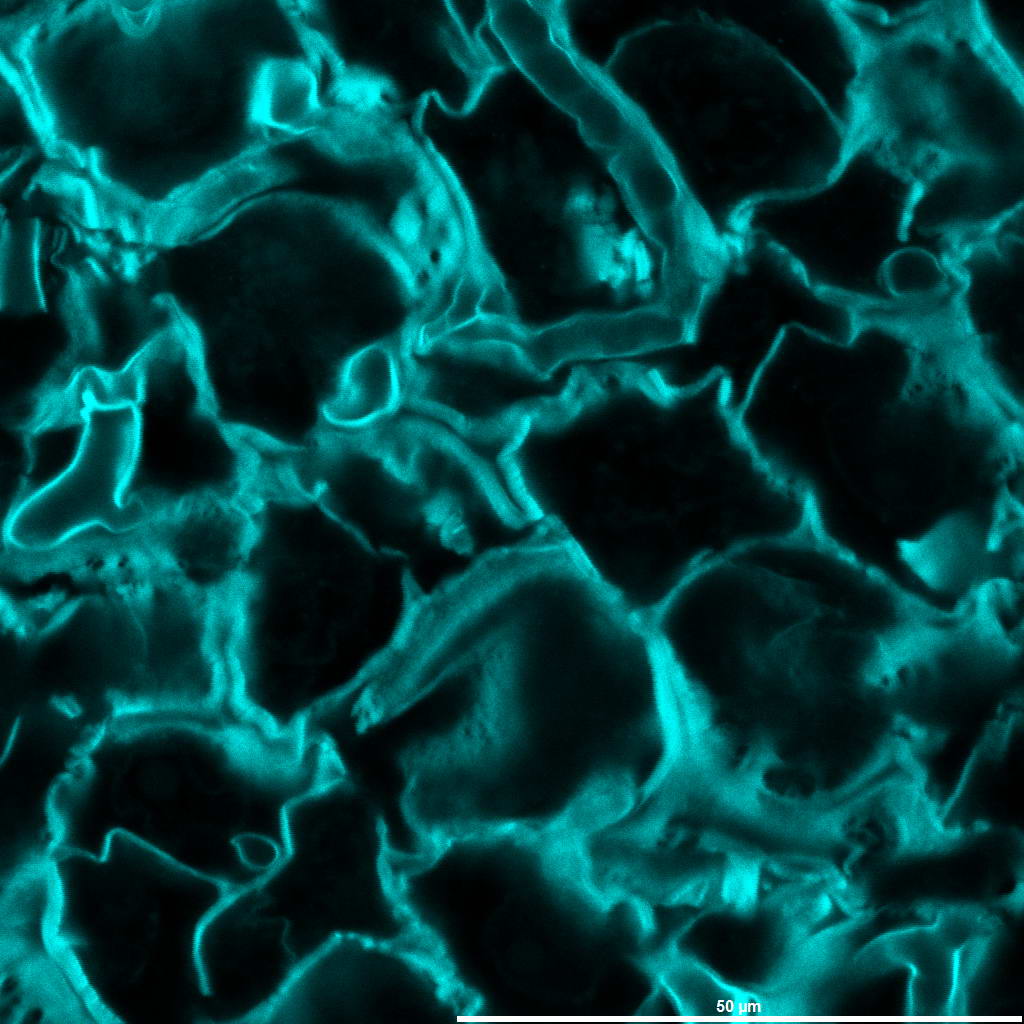

Supplement: Supplementary file 1 [file plants-14-01083-s001.zip › Appendix_A-CLSM-set-images_Fig8/Tfrezzi_peanut_CalcW_Ser09_z13.jpg]

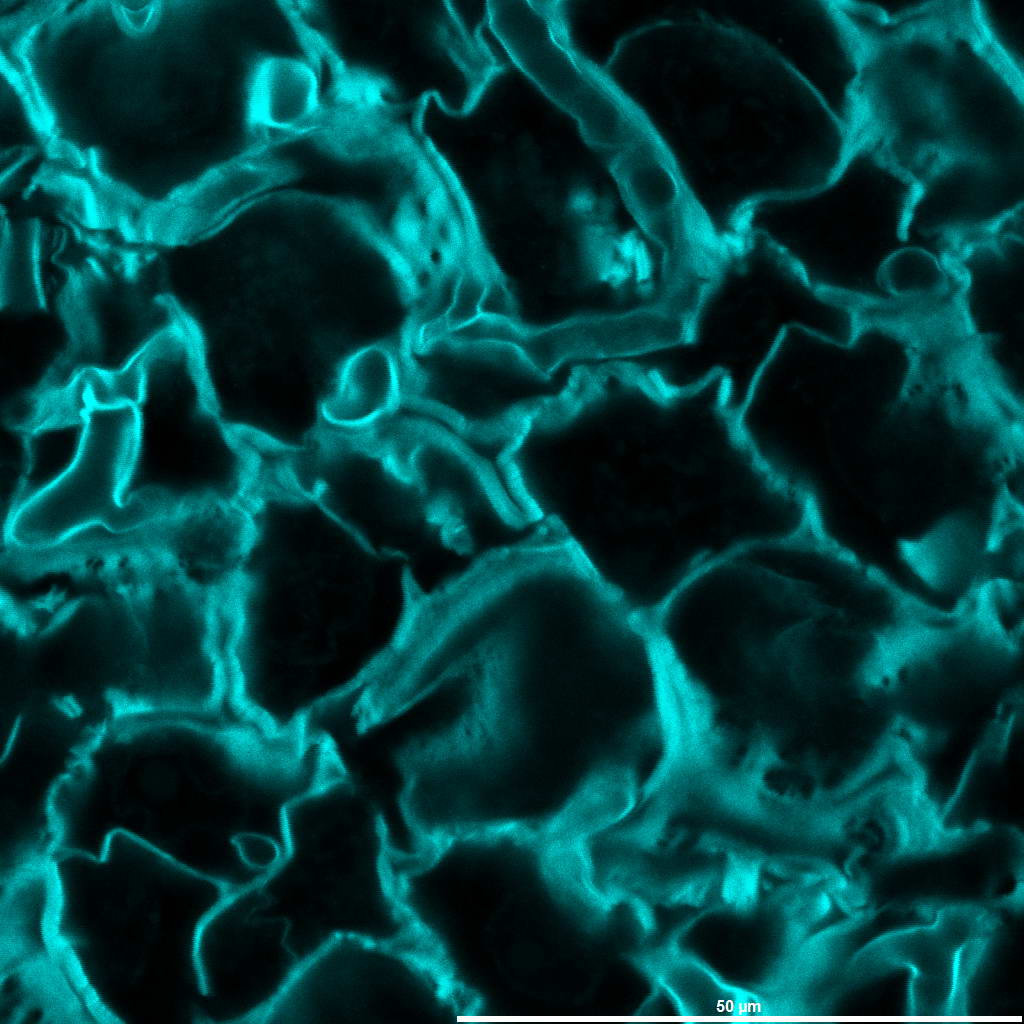

Supplement: Supplementary file 1 [file plants-14-01083-s001.zip › Appendix_A-CLSM-set-images_Fig8/Tfrezzi_peanut_CalcW_Ser09_z14.jpg]

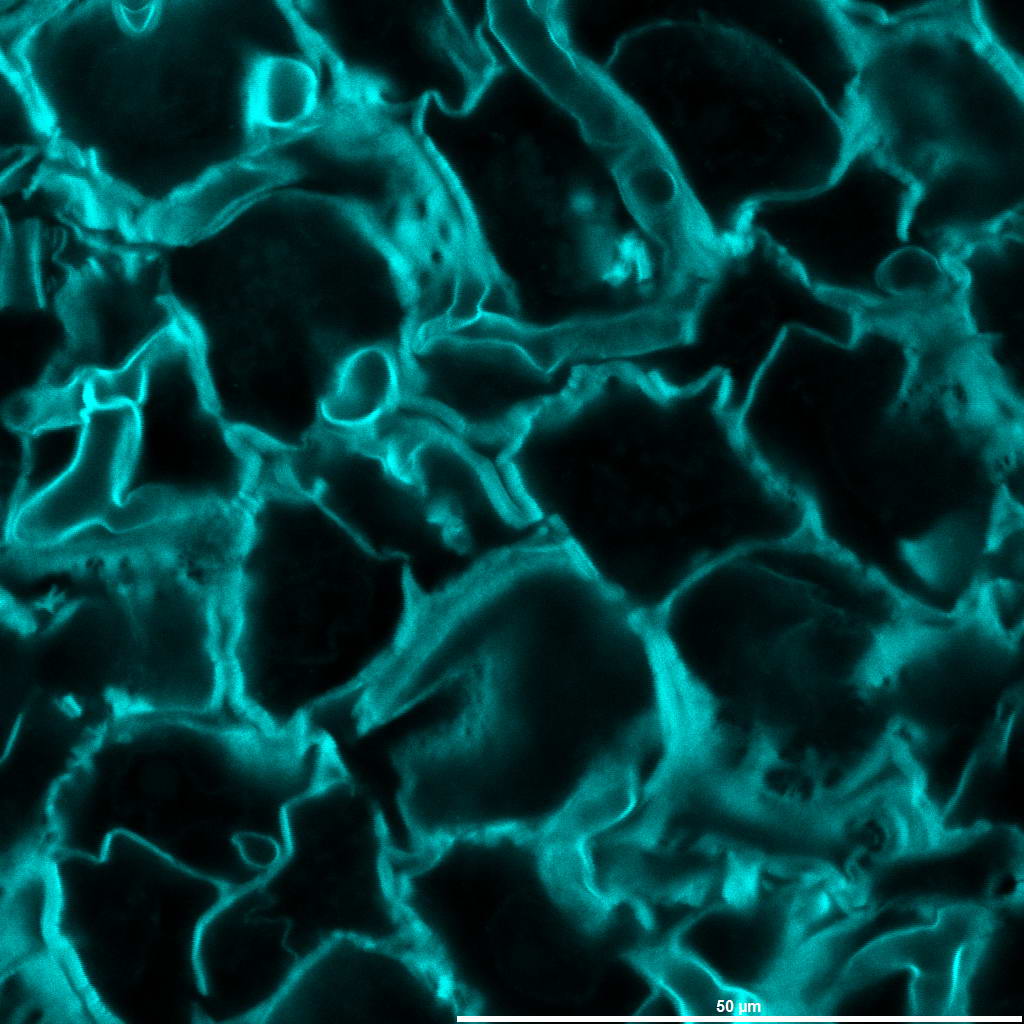

Supplement: Supplementary file 1 [file plants-14-01083-s001.zip › Appendix_A-CLSM-set-images_Fig8/Tfrezzi_peanut_CalcW_Ser09_z15.jpg]

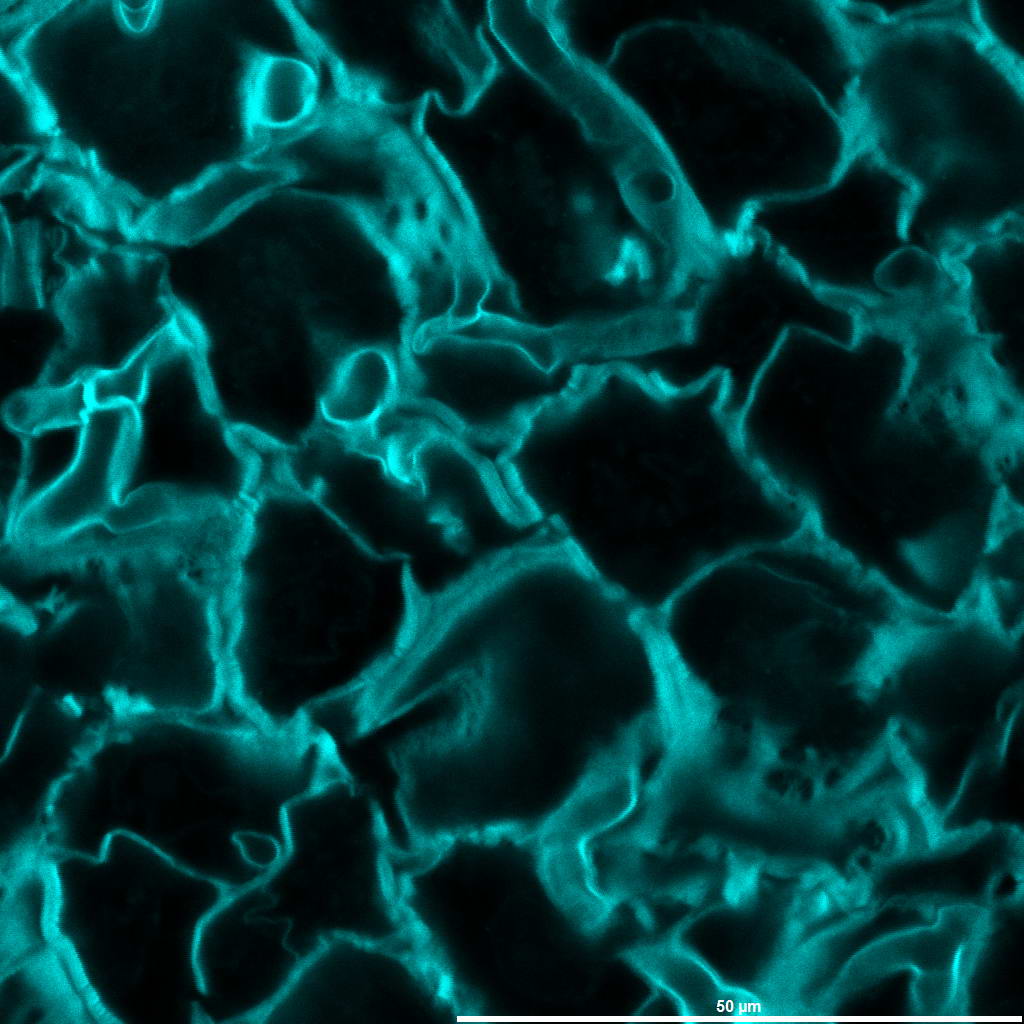

Supplement: Supplementary file 1 [file plants-14-01083-s001.zip › Appendix_A-CLSM-set-images_Fig8/Tfrezzi_peanut_CalcW_Ser09_z16.jpg]

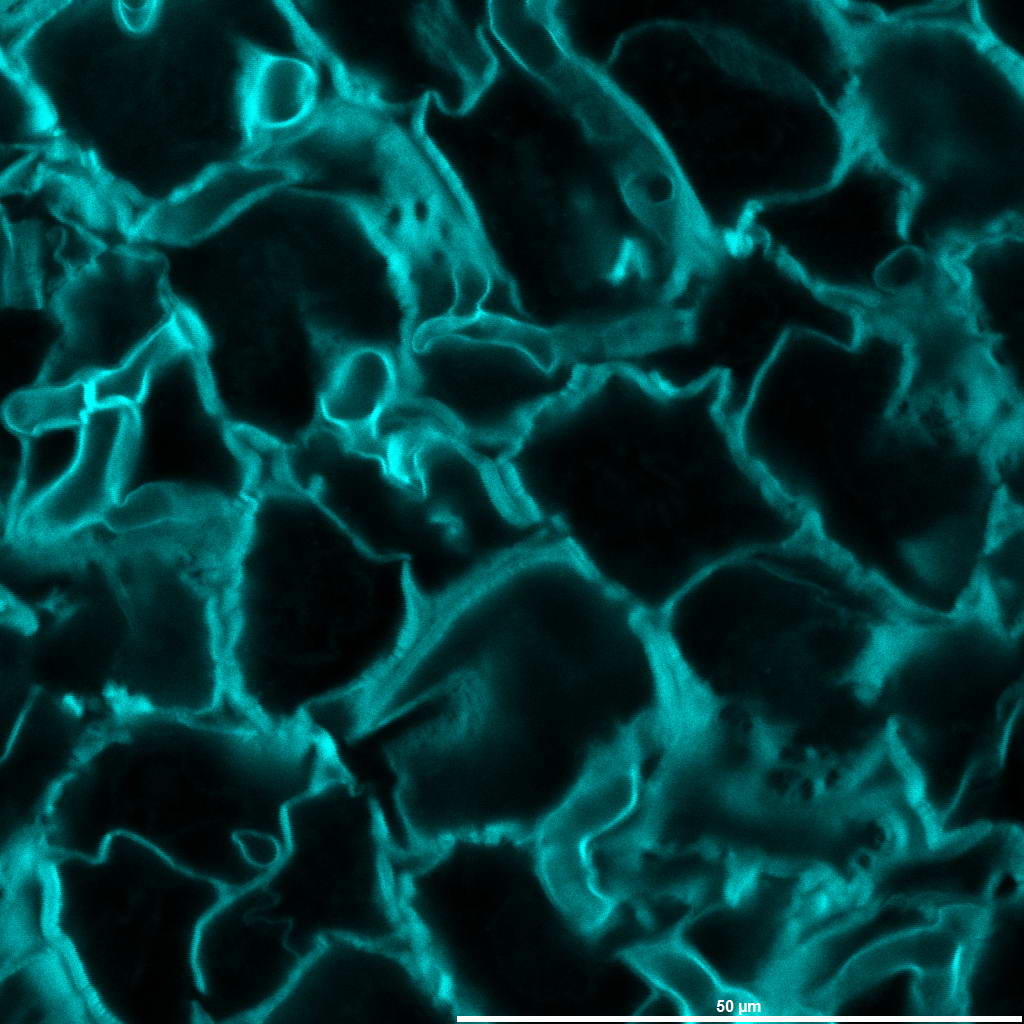

Supplement: Supplementary file 1 [file plants-14-01083-s001.zip › Appendix_A-CLSM-set-images_Fig8/Tfrezzi_peanut_CalcW_Ser09_z17.jpg]

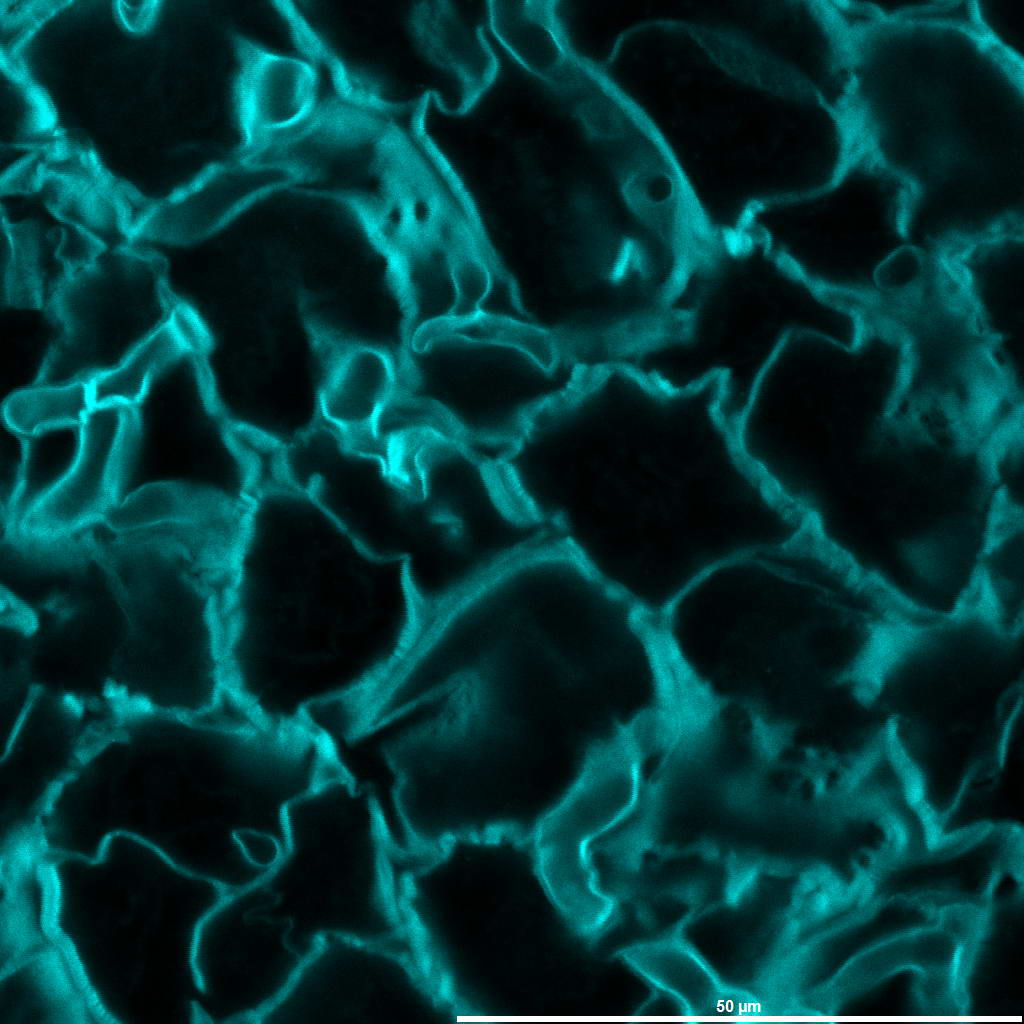

Supplement: Supplementary file 1 [file plants-14-01083-s001.zip › Appendix_A-CLSM-set-images_Fig8/Tfrezzi_peanut_CalcW_Ser09_z18.jpg]

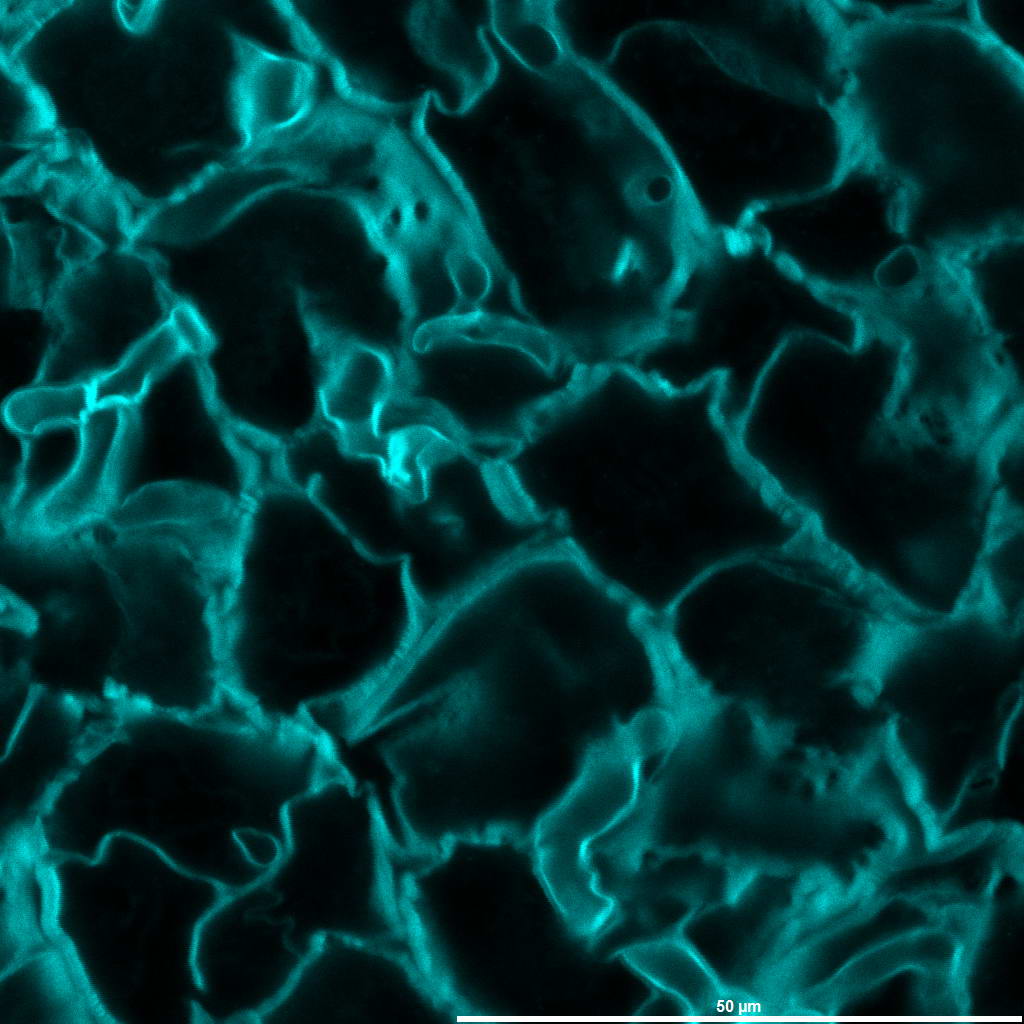

Supplement: Supplementary file 1 [file plants-14-01083-s001.zip › Appendix_A-CLSM-set-images_Fig8/Tfrezzi_peanut_CalcW_Ser09_z19.jpg]

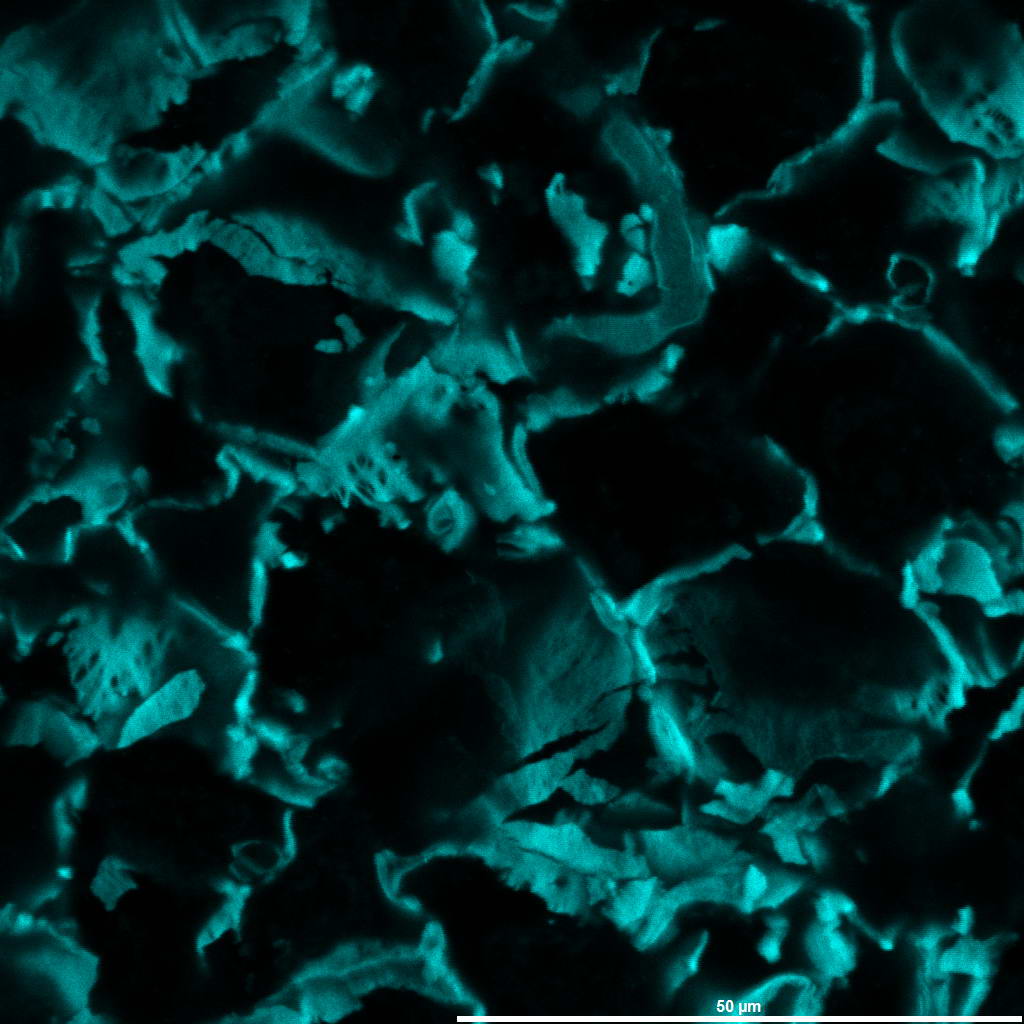

Supplement: Supplementary file 1 [file plants-14-01083-s001.zip › Appendix_A-CLSM-set-images_Fig8/Tfrezzi_peanut_CalcW_Ser09_z2.jpg]

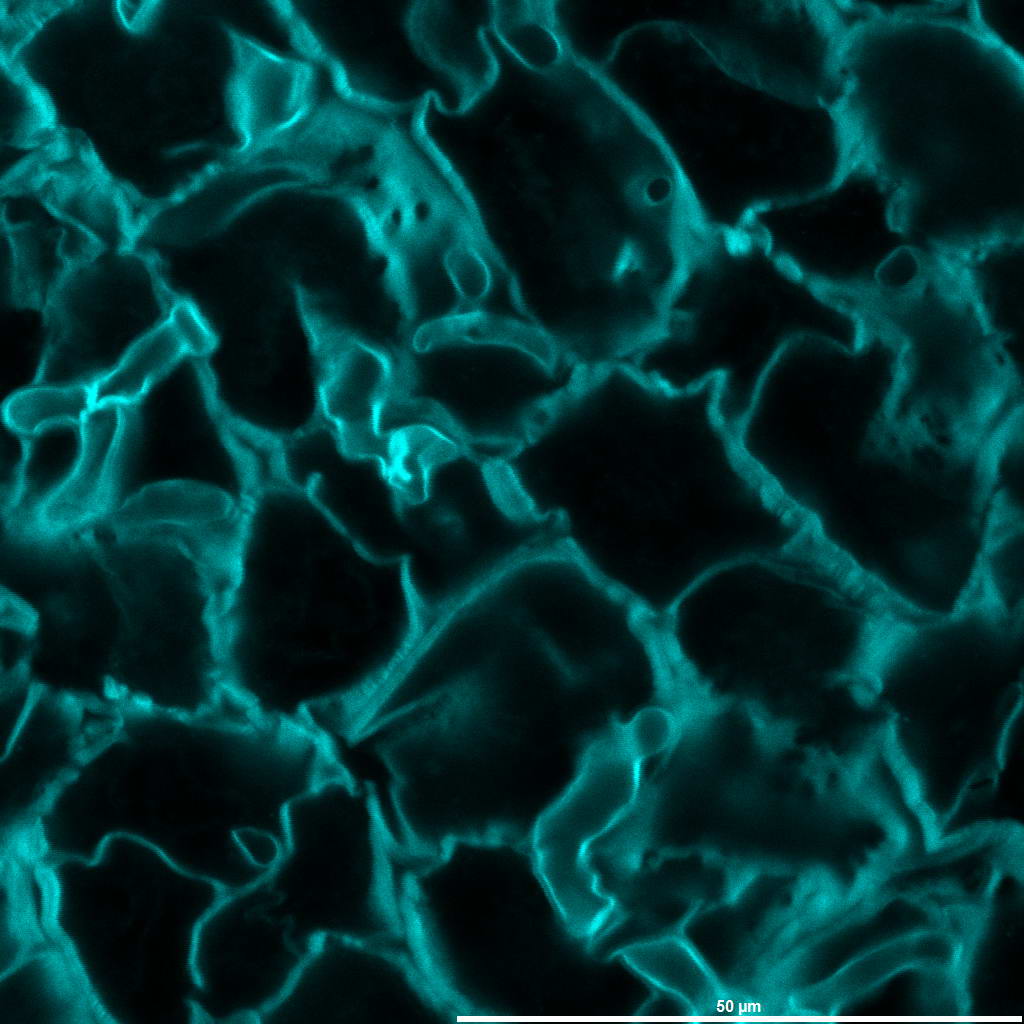

Supplement: Supplementary file 1 [file plants-14-01083-s001.zip › Appendix_A-CLSM-set-images_Fig8/Tfrezzi_peanut_CalcW_Ser09_z20.jpg]

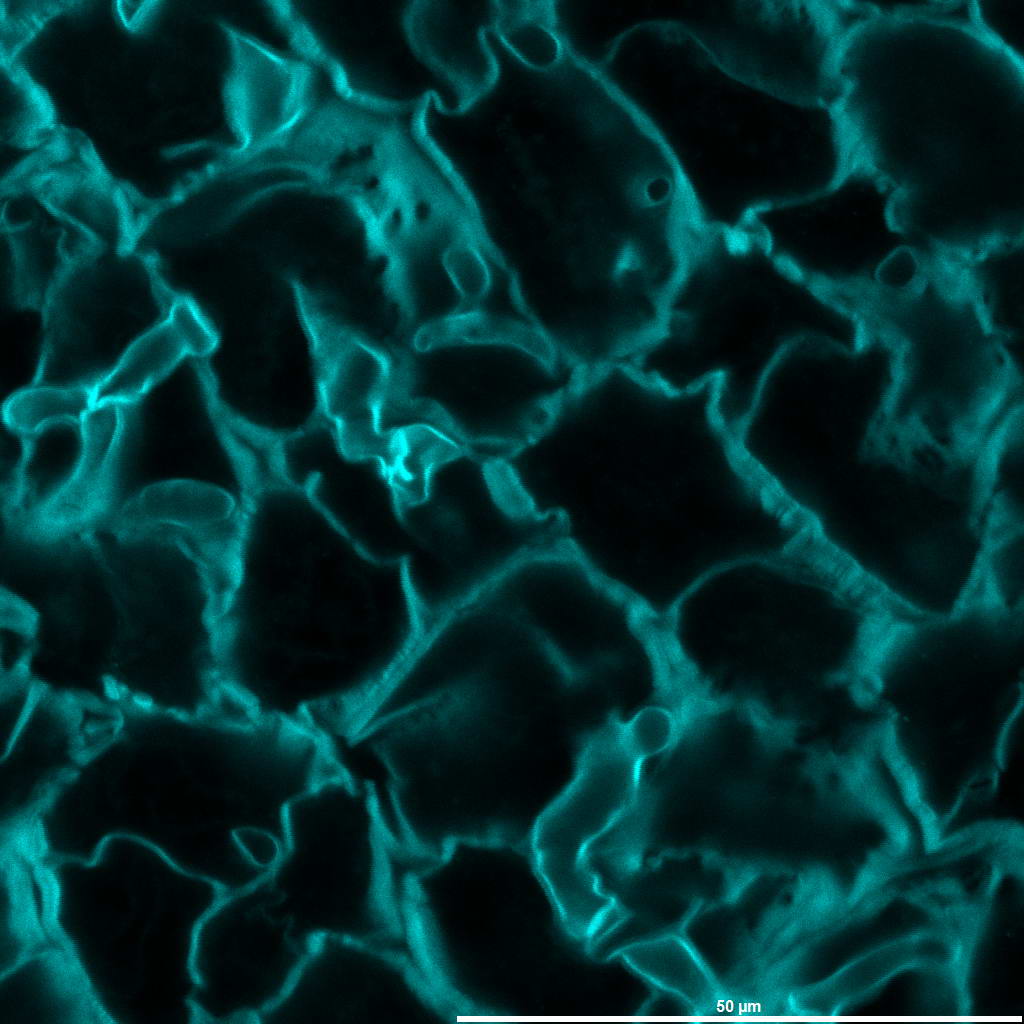

Supplement: Supplementary file 1 [file plants-14-01083-s001.zip › Appendix_A-CLSM-set-images_Fig8/Tfrezzi_peanut_CalcW_Ser09_z21.jpg]

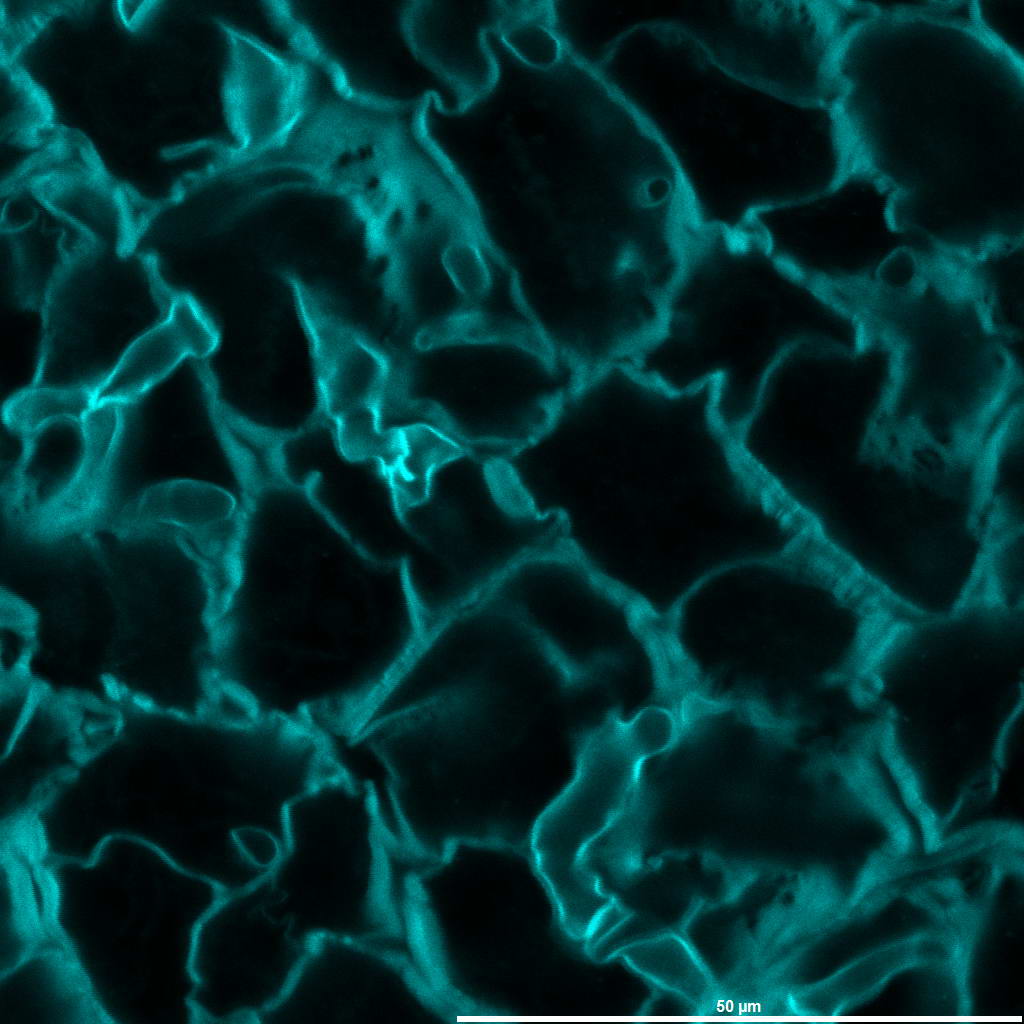

Supplement: Supplementary file 1 [file plants-14-01083-s001.zip › Appendix_A-CLSM-set-images_Fig8/Tfrezzi_peanut_CalcW_Ser09_z22.jpg]

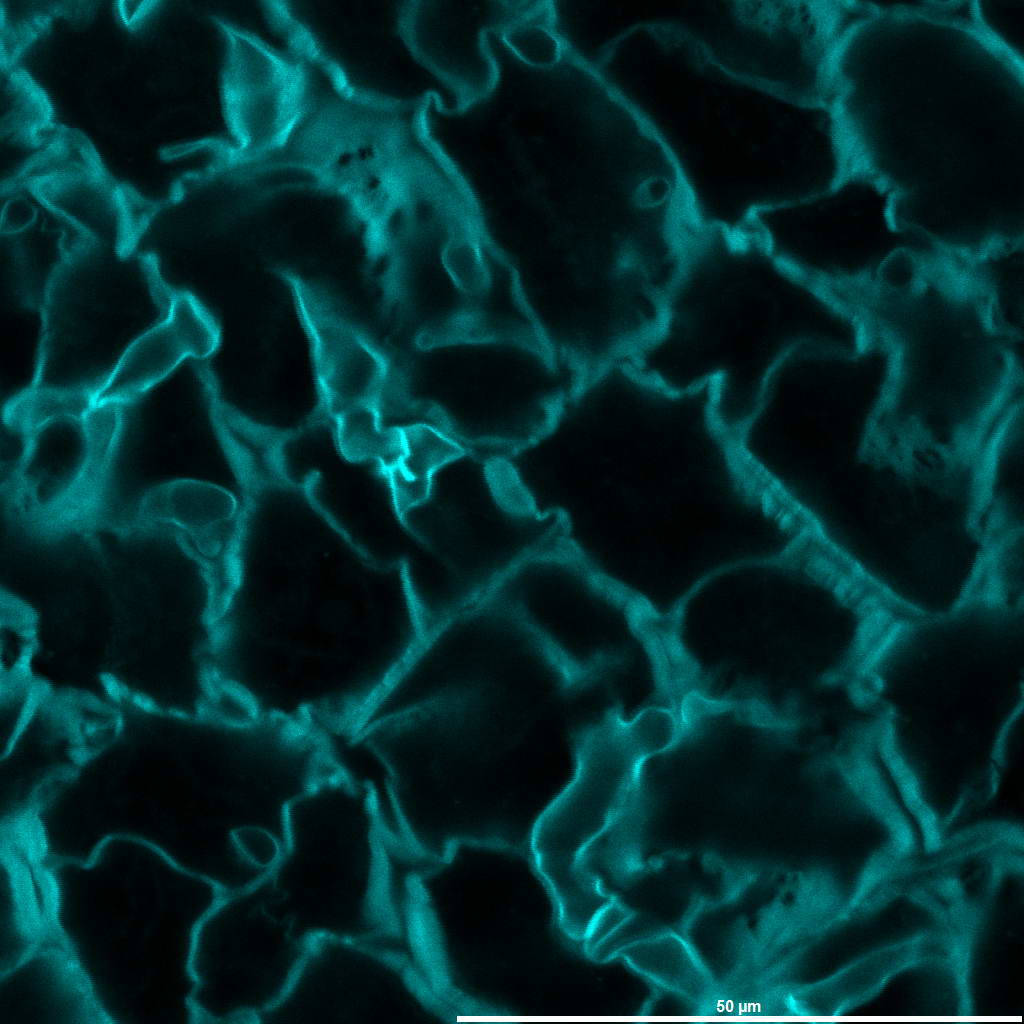

Supplement: Supplementary file 1 [file plants-14-01083-s001.zip › Appendix_A-CLSM-set-images_Fig8/Tfrezzi_peanut_CalcW_Ser09_z23.jpg]

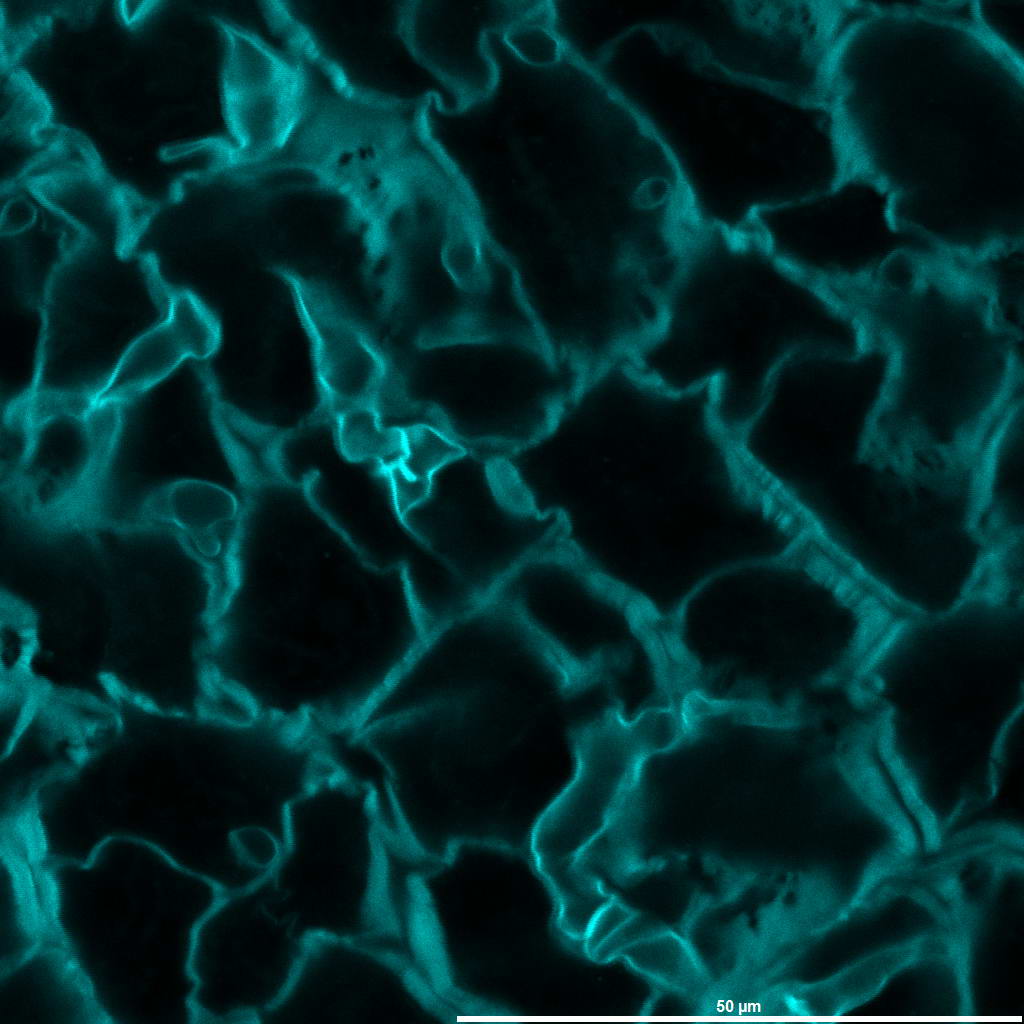

Supplement: Supplementary file 1 [file plants-14-01083-s001.zip › Appendix_A-CLSM-set-images_Fig8/Tfrezzi_peanut_CalcW_Ser09_z24.jpg]

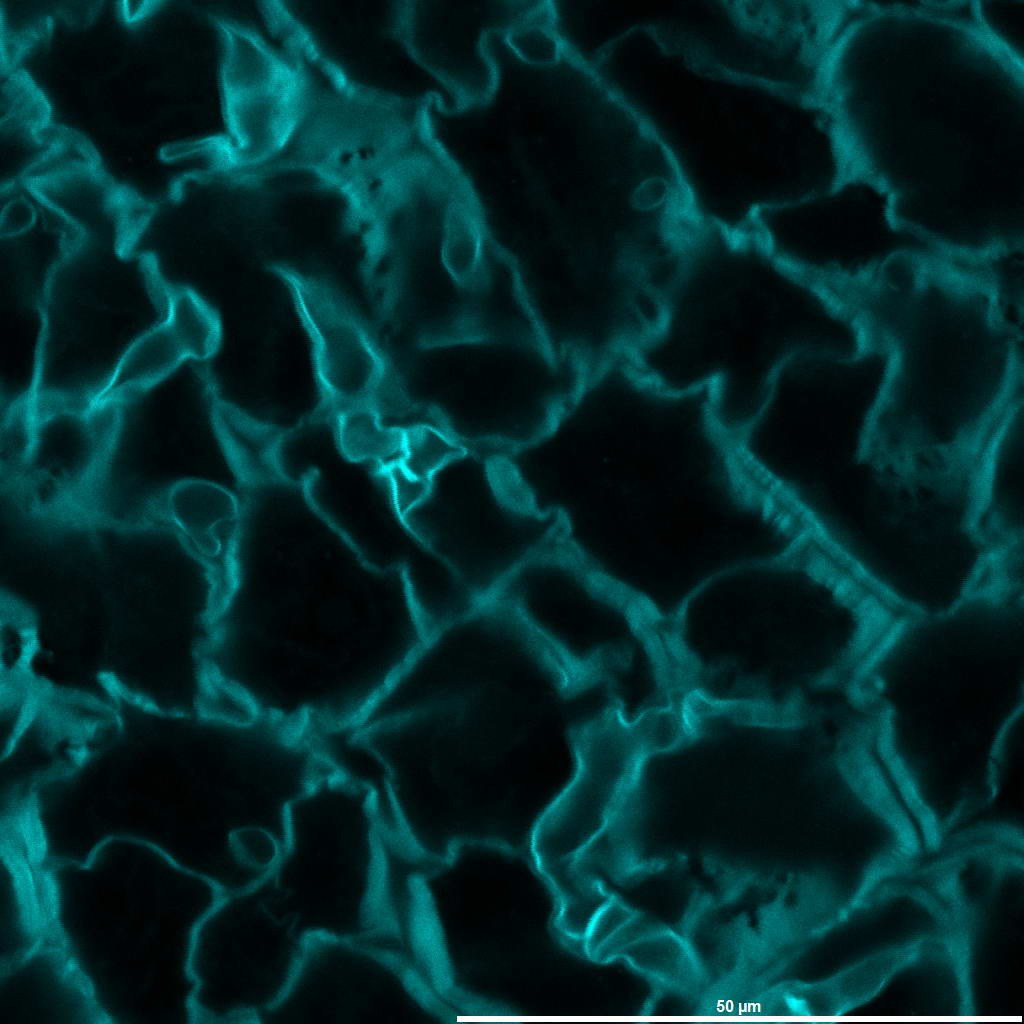

Supplement: Supplementary file 1 [file plants-14-01083-s001.zip › Appendix_A-CLSM-set-images_Fig8/Tfrezzi_peanut_CalcW_Ser09_z25.jpg]

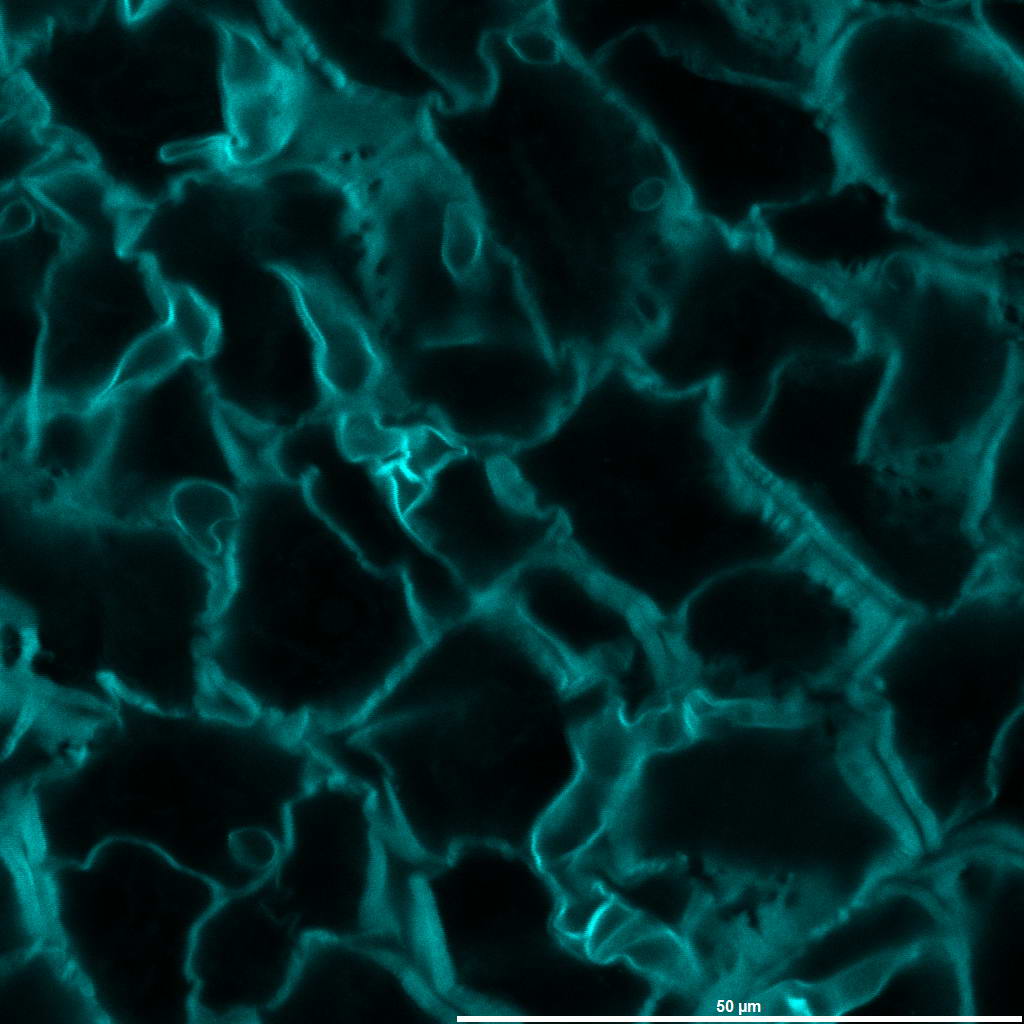

Supplement: Supplementary file 1 [file plants-14-01083-s001.zip › Appendix_A-CLSM-set-images_Fig8/Tfrezzi_peanut_CalcW_Ser09_z26.jpg]

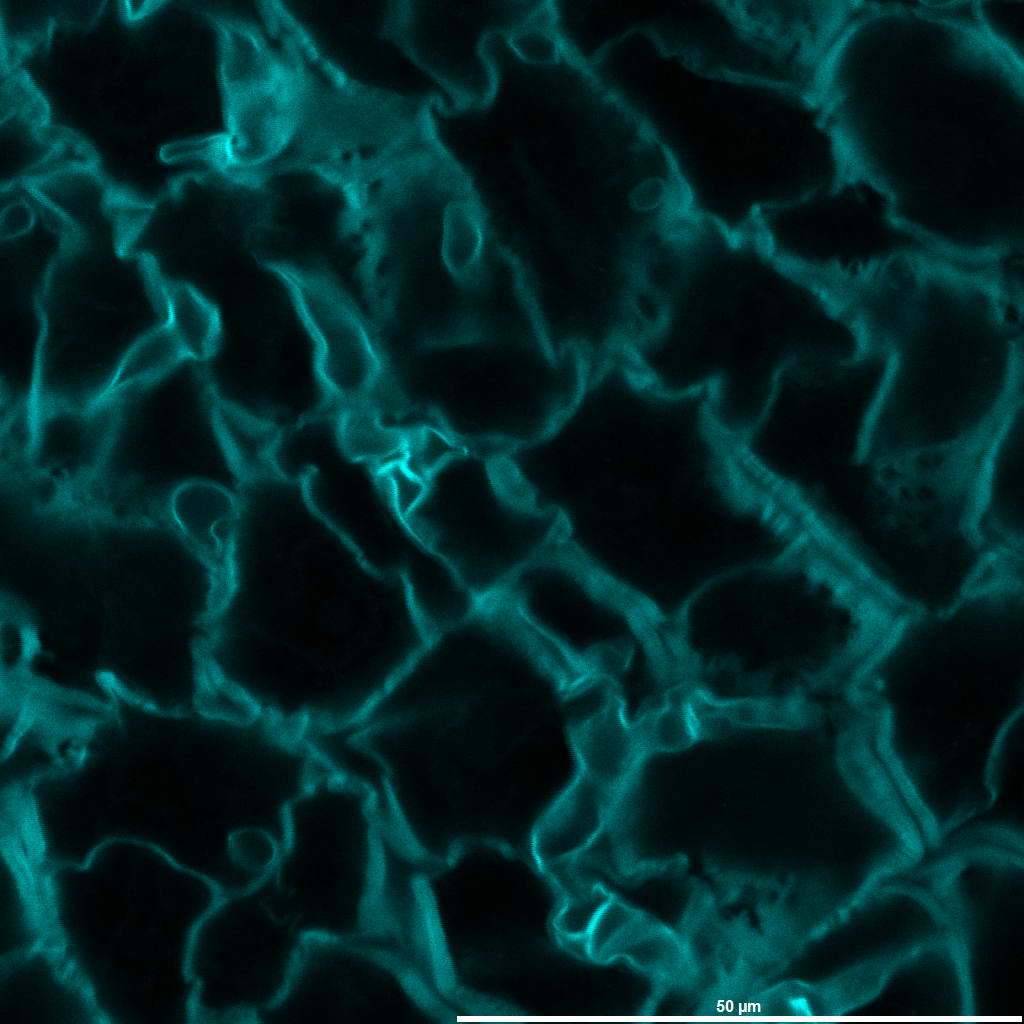

Supplement: Supplementary file 1 [file plants-14-01083-s001.zip › Appendix_A-CLSM-set-images_Fig8/Tfrezzi_peanut_CalcW_Ser09_z27.jpg]

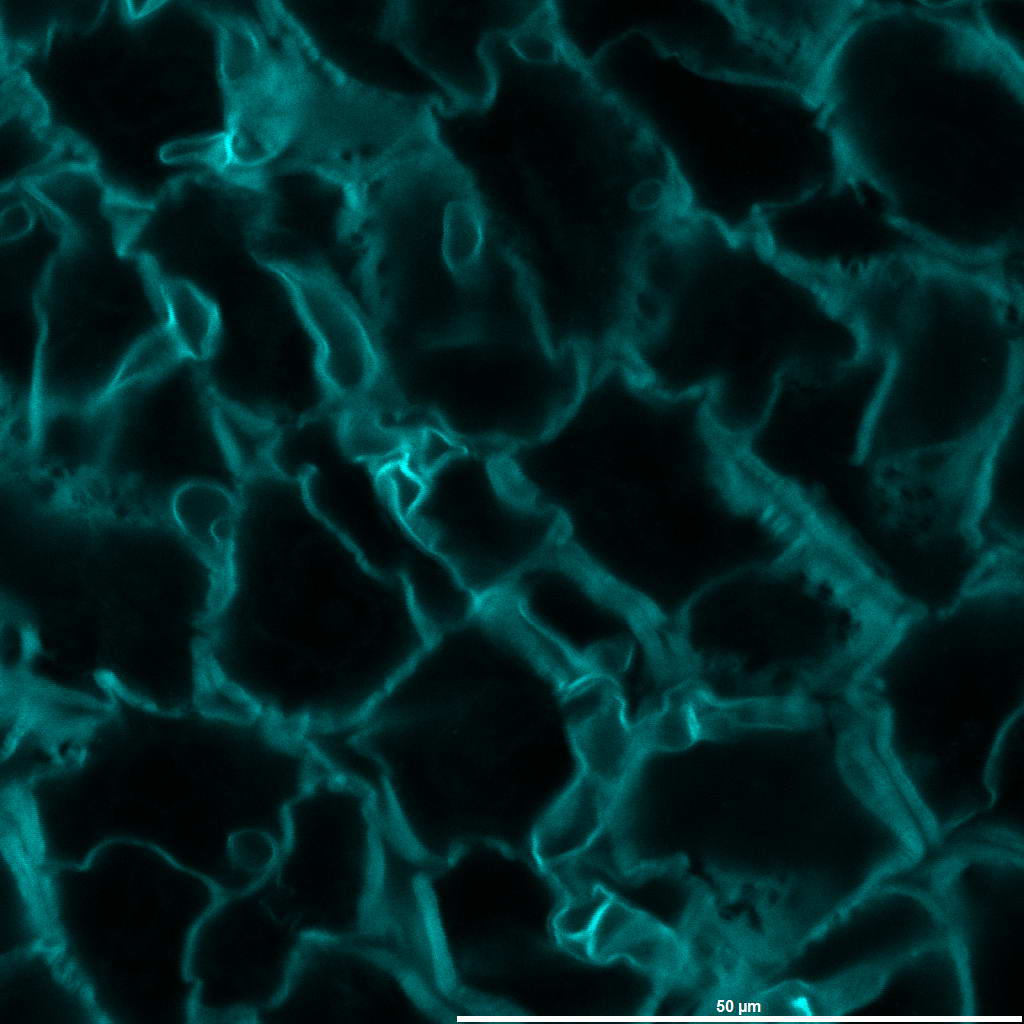

Supplement: Supplementary file 1 [file plants-14-01083-s001.zip › Appendix_A-CLSM-set-images_Fig8/Tfrezzi_peanut_CalcW_Ser09_z28.jpg]

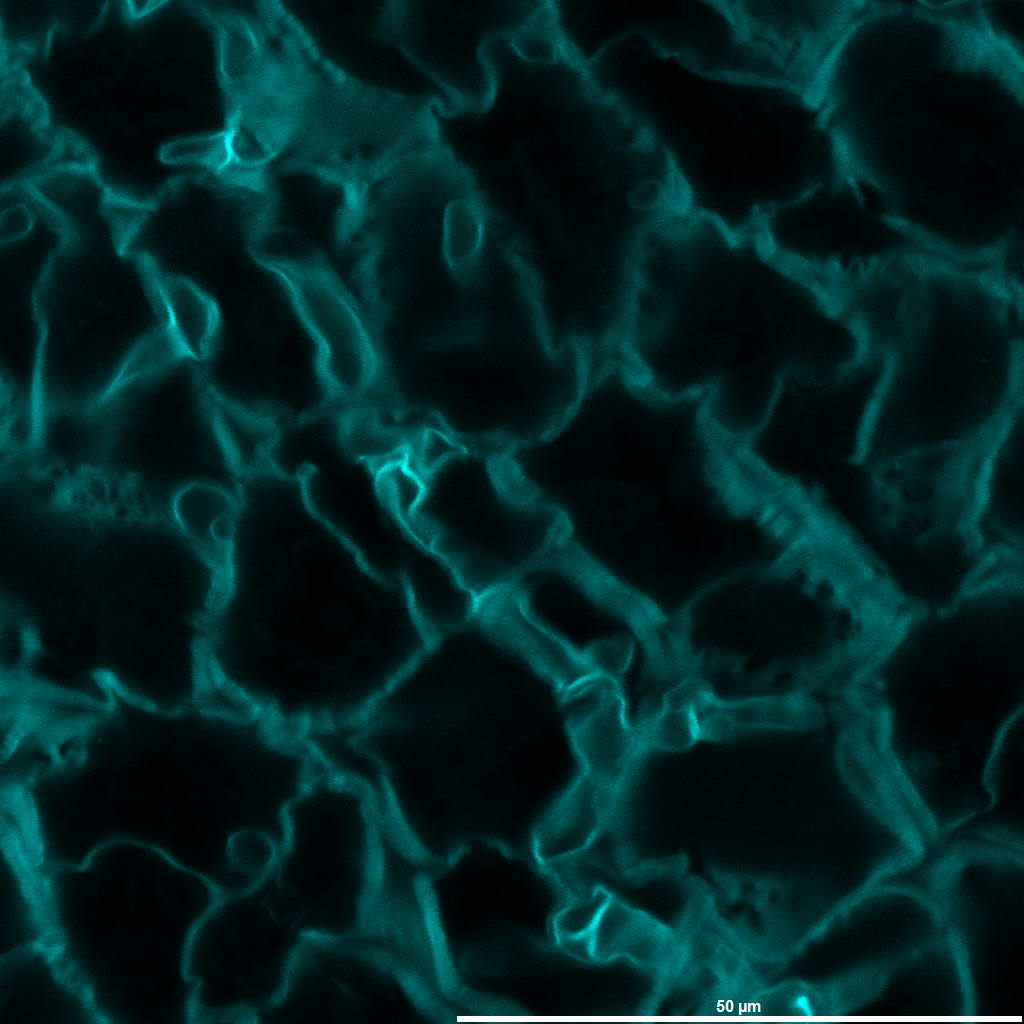

Supplement: Supplementary file 1 [file plants-14-01083-s001.zip › Appendix_A-CLSM-set-images_Fig8/Tfrezzi_peanut_CalcW_Ser09_z29.jpg]

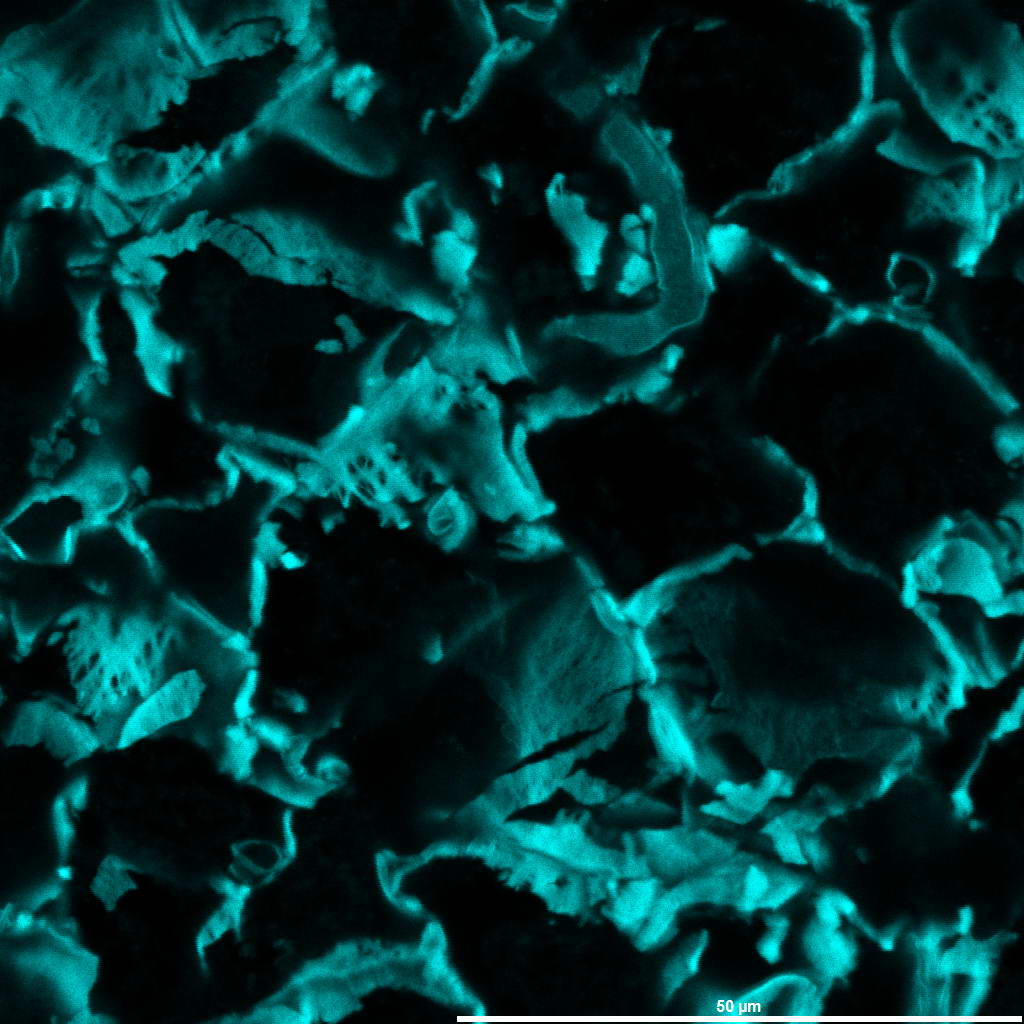

Supplement: Supplementary file 1 [file plants-14-01083-s001.zip › Appendix_A-CLSM-set-images_Fig8/Tfrezzi_peanut_CalcW_Ser09_z3.jpg]

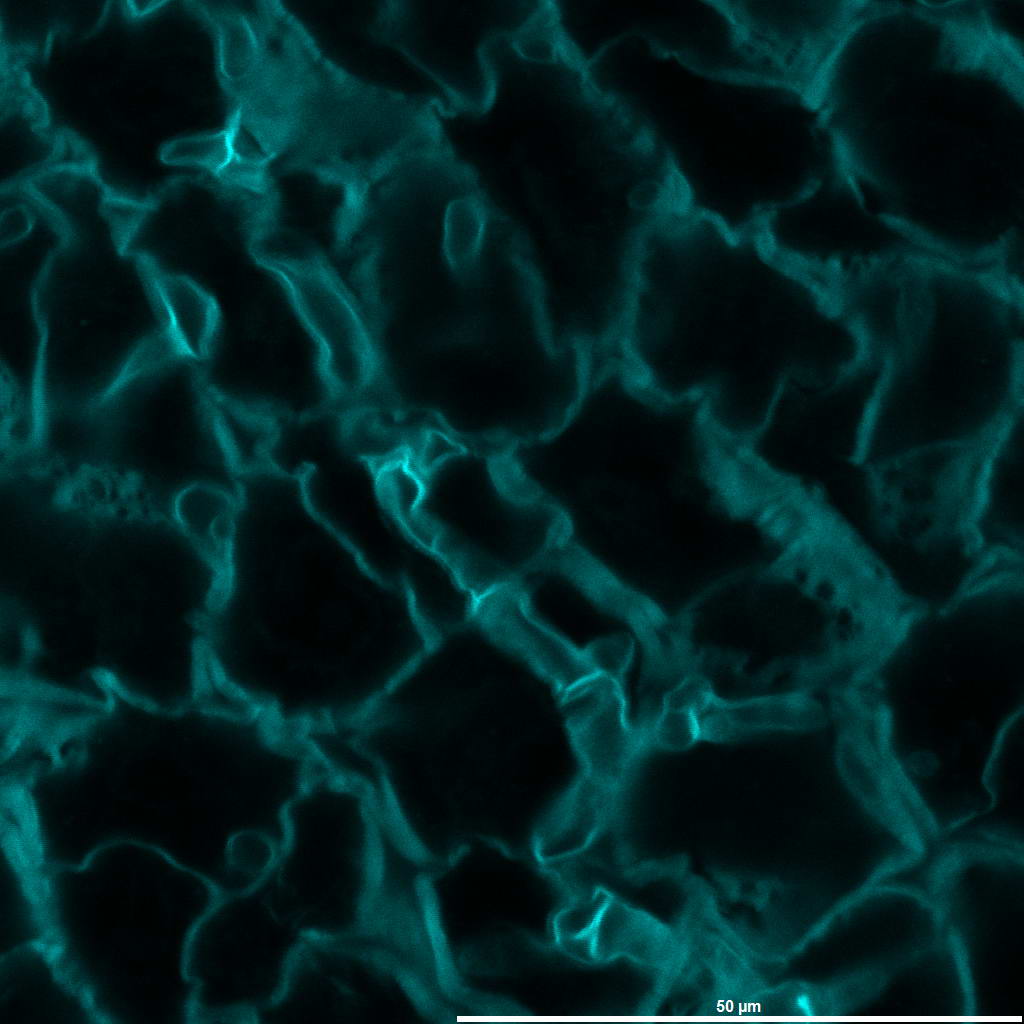

Supplement: Supplementary file 1 [file plants-14-01083-s001.zip › Appendix_A-CLSM-set-images_Fig8/Tfrezzi_peanut_CalcW_Ser09_z30.jpg]

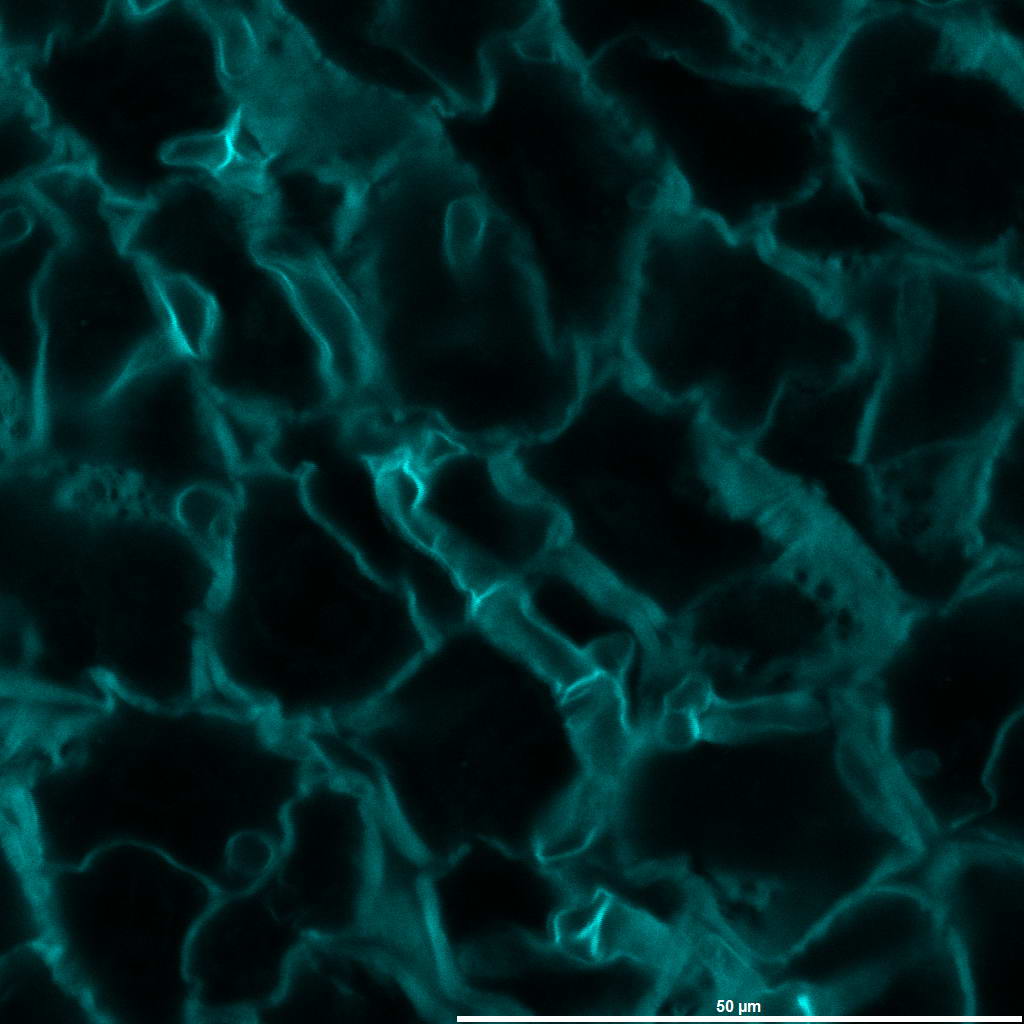

Supplement: Supplementary file 1 [file plants-14-01083-s001.zip › Appendix_A-CLSM-set-images_Fig8/Tfrezzi_peanut_CalcW_Ser09_z31.jpg]

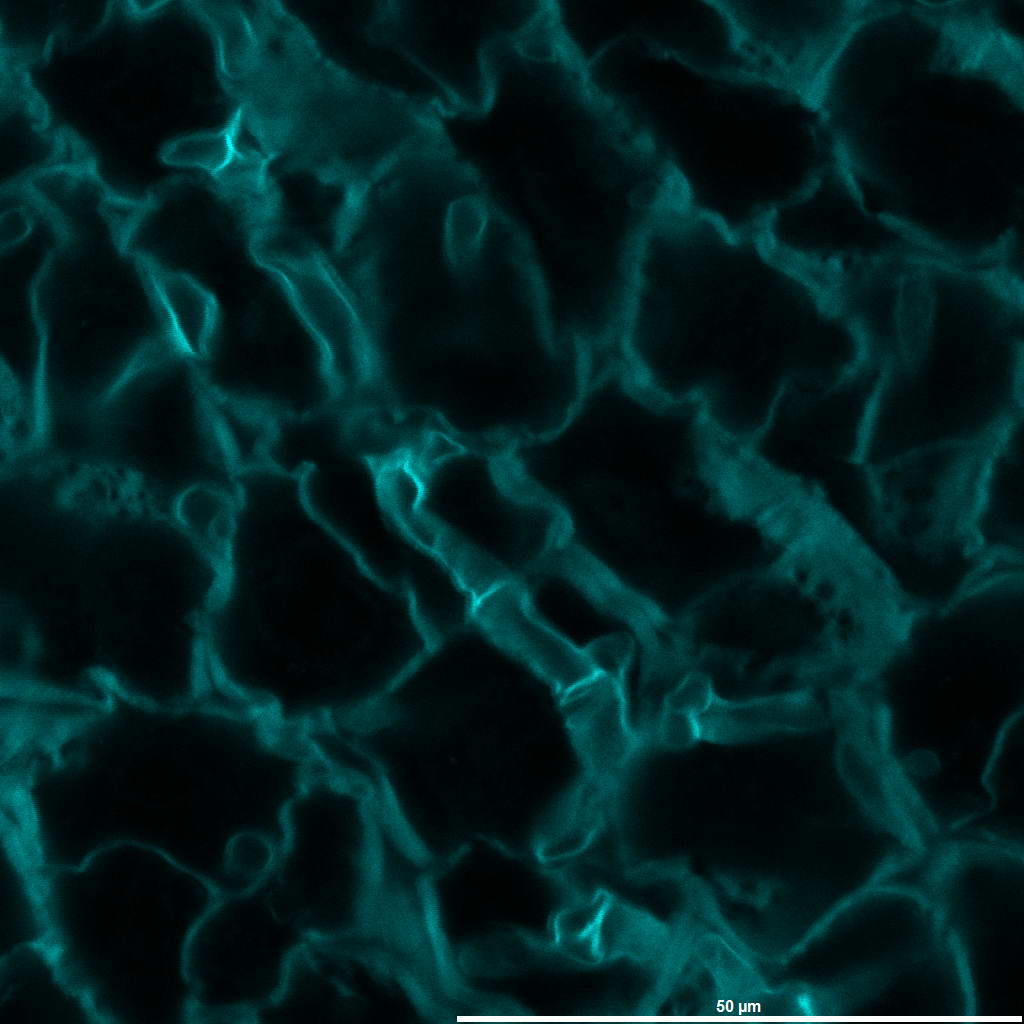

Supplement: Supplementary file 1 [file plants-14-01083-s001.zip › Appendix_A-CLSM-set-images_Fig8/Tfrezzi_peanut_CalcW_Ser09_z32.jpg]

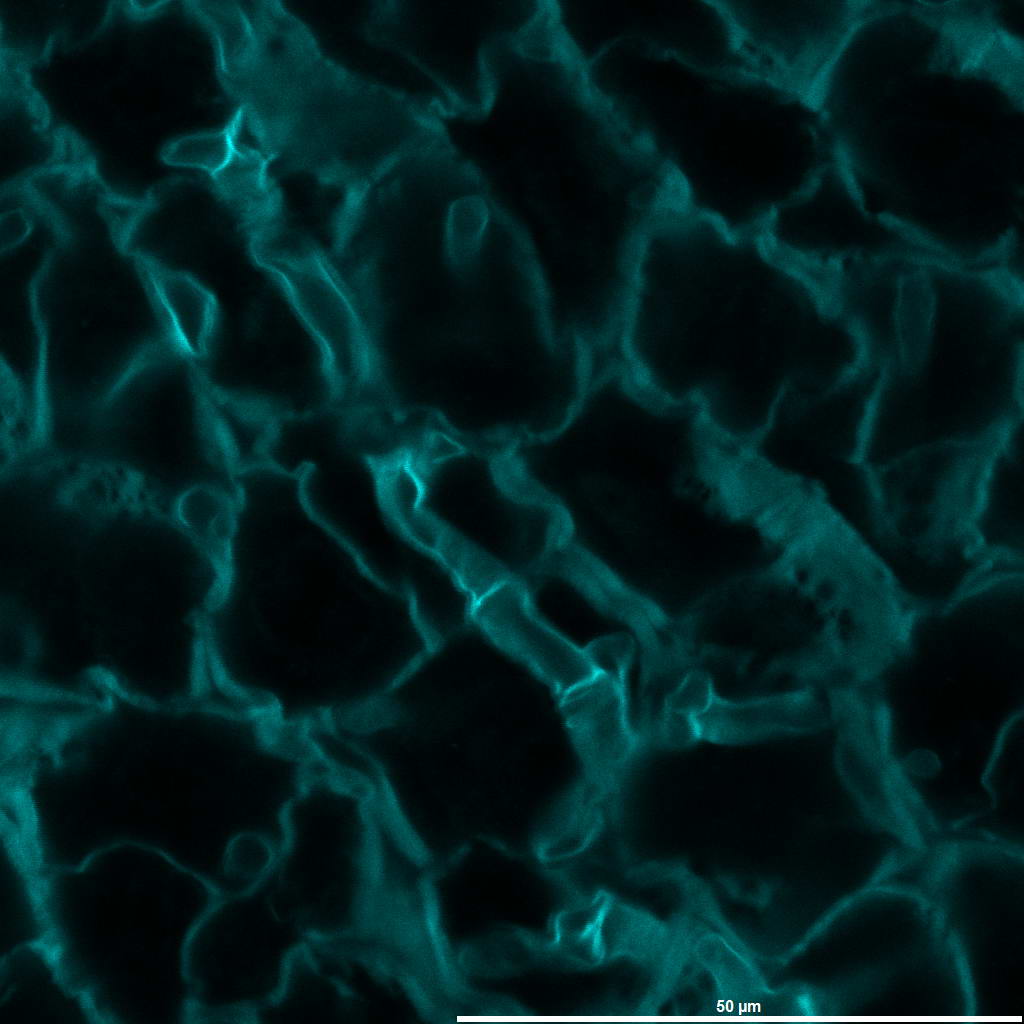

Supplement: Supplementary file 1 [file plants-14-01083-s001.zip › Appendix_A-CLSM-set-images_Fig8/Tfrezzi_peanut_CalcW_Ser09_z33.jpg]

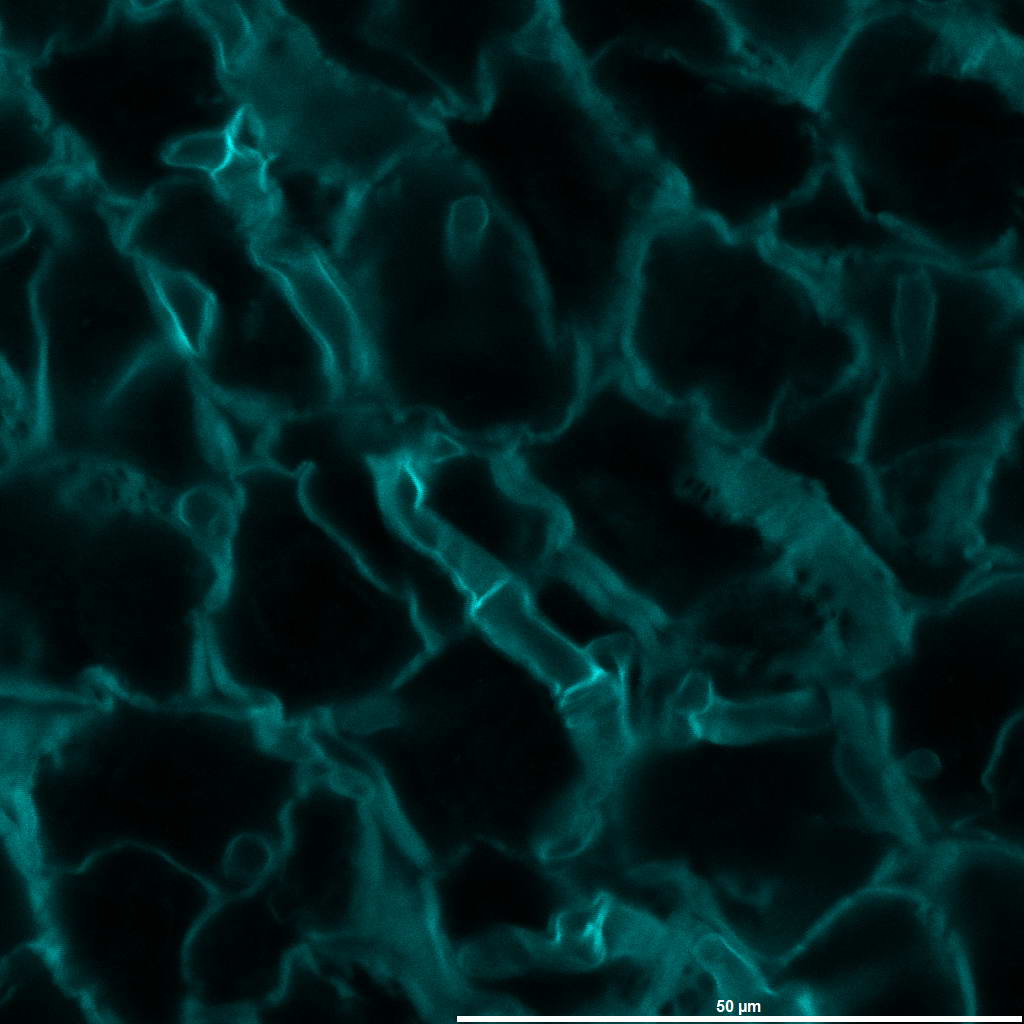

Supplement: Supplementary file 1 [file plants-14-01083-s001.zip › Appendix_A-CLSM-set-images_Fig8/Tfrezzi_peanut_CalcW_Ser09_z34.jpg]

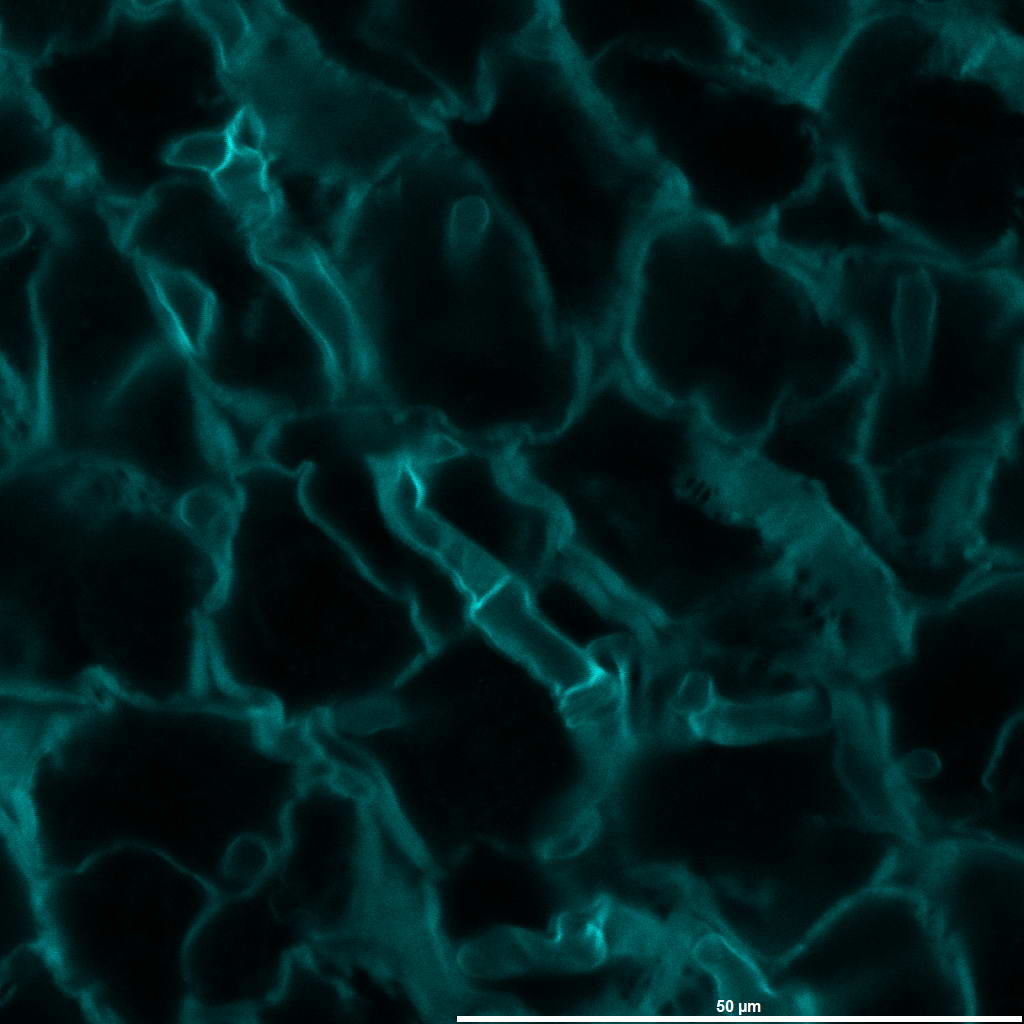

Supplement: Supplementary file 1 [file plants-14-01083-s001.zip › Appendix_A-CLSM-set-images_Fig8/Tfrezzi_peanut_CalcW_Ser09_z35.jpg]

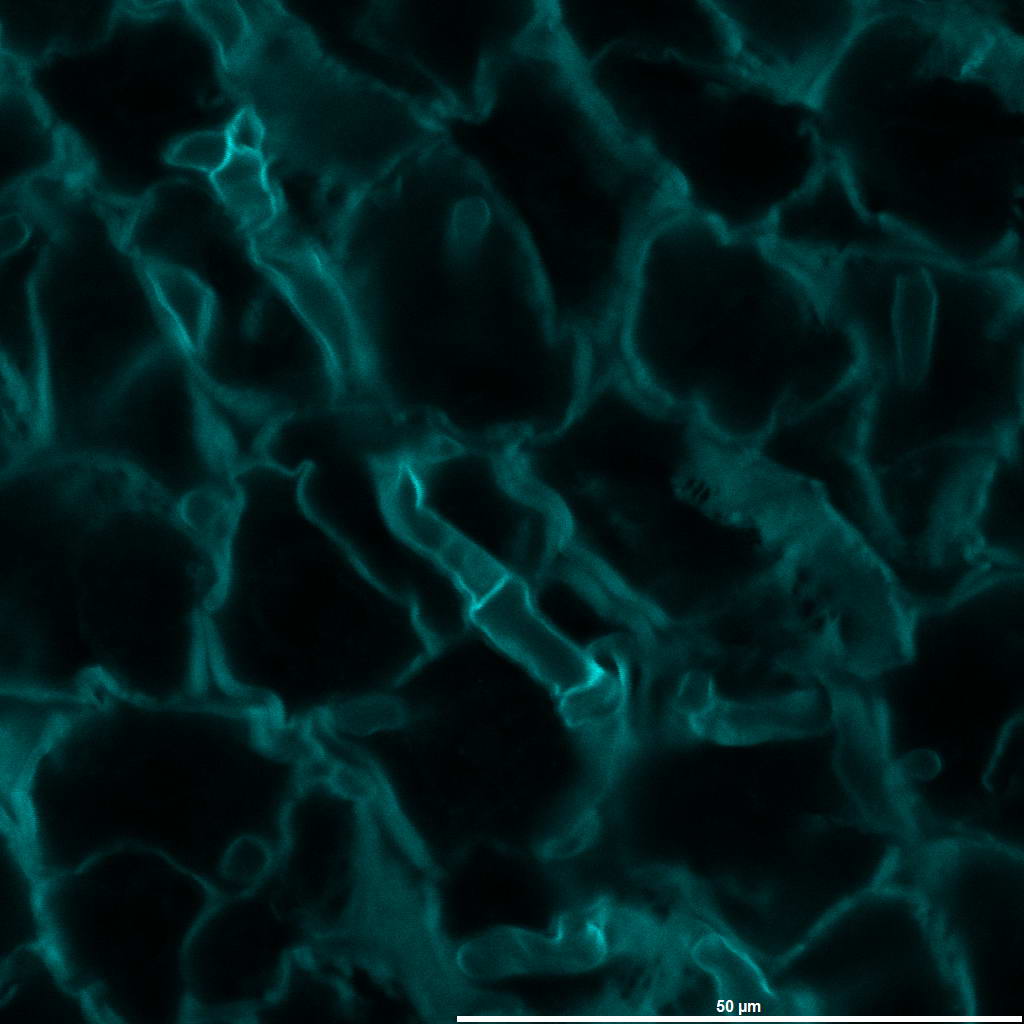

Supplement: Supplementary file 1 [file plants-14-01083-s001.zip › Appendix_A-CLSM-set-images_Fig8/Tfrezzi_peanut_CalcW_Ser09_z36.jpg]

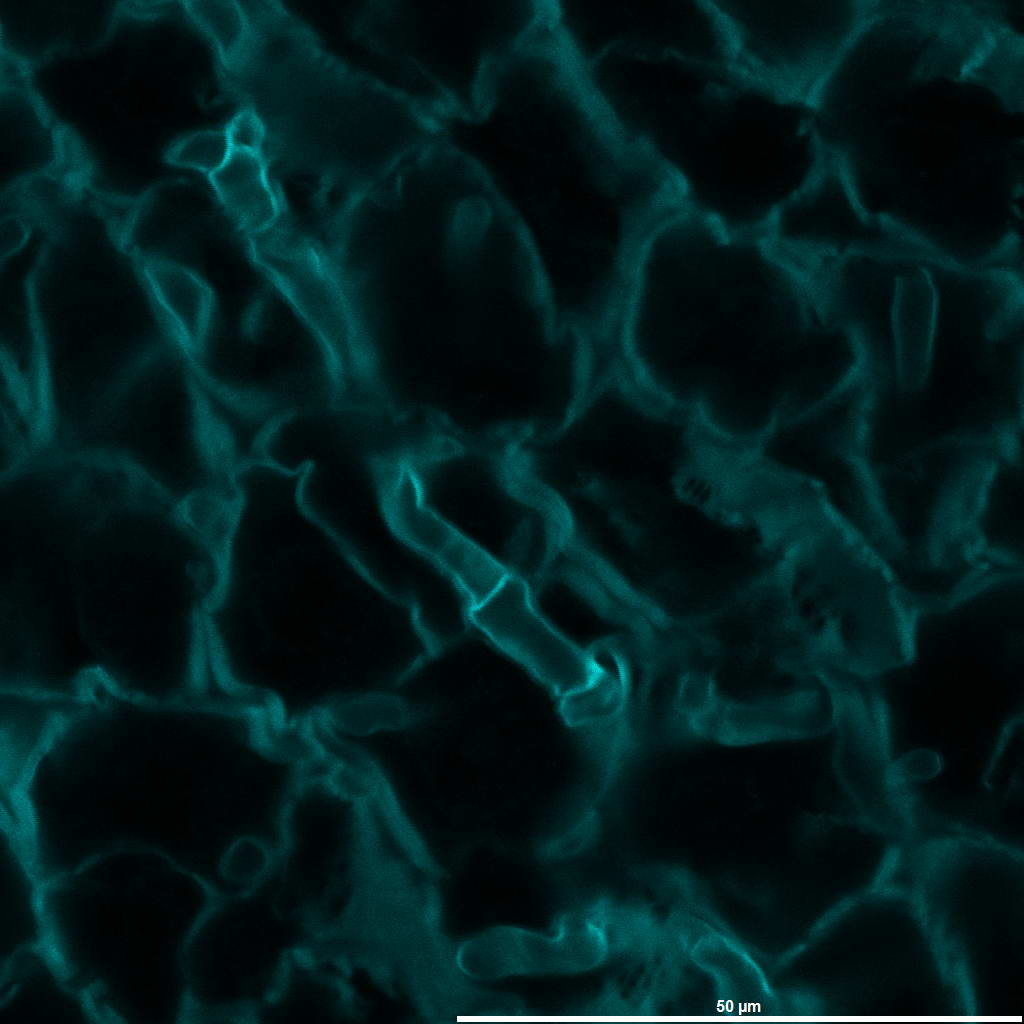

Supplement: Supplementary file 1 [file plants-14-01083-s001.zip › Appendix_A-CLSM-set-images_Fig8/Tfrezzi_peanut_CalcW_Ser09_z37.jpg]

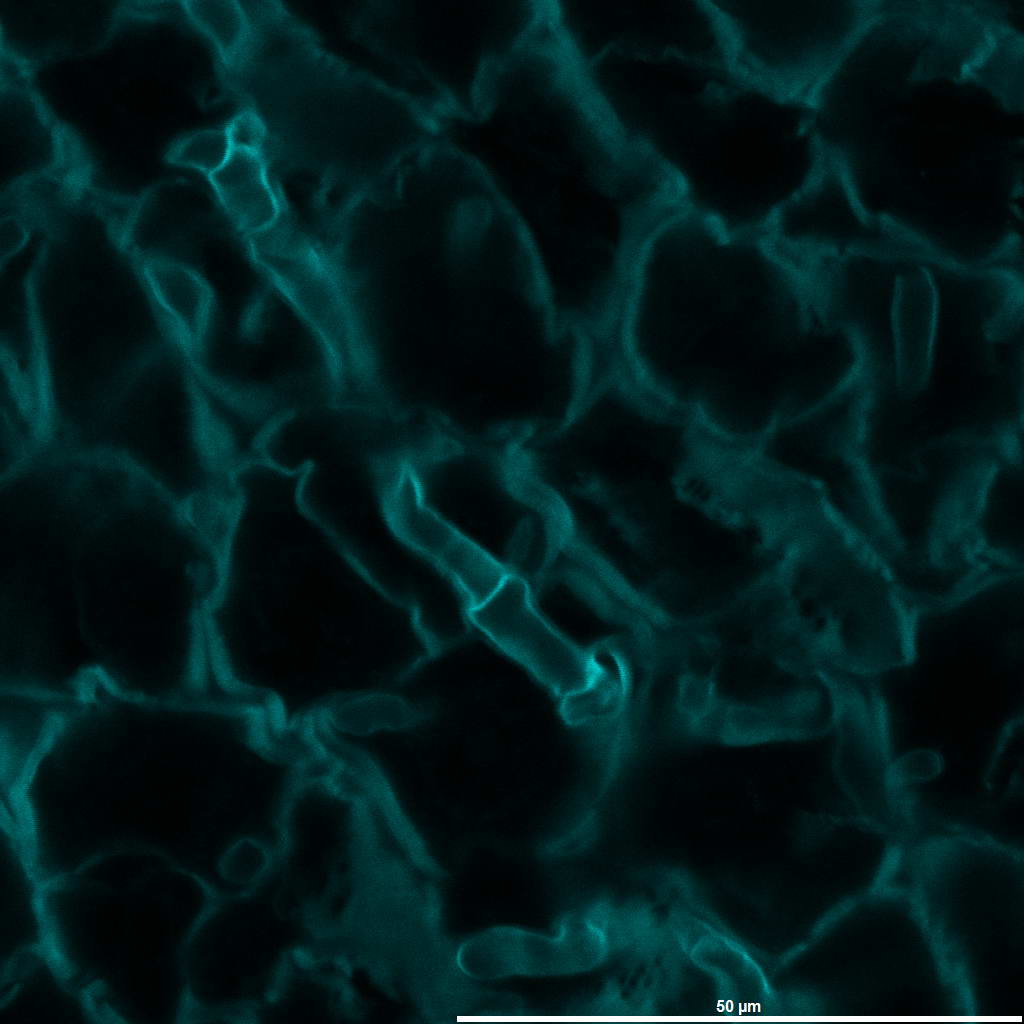

Supplement: Supplementary file 1 [file plants-14-01083-s001.zip › Appendix_A-CLSM-set-images_Fig8/Tfrezzi_peanut_CalcW_Ser09_z38.jpg]

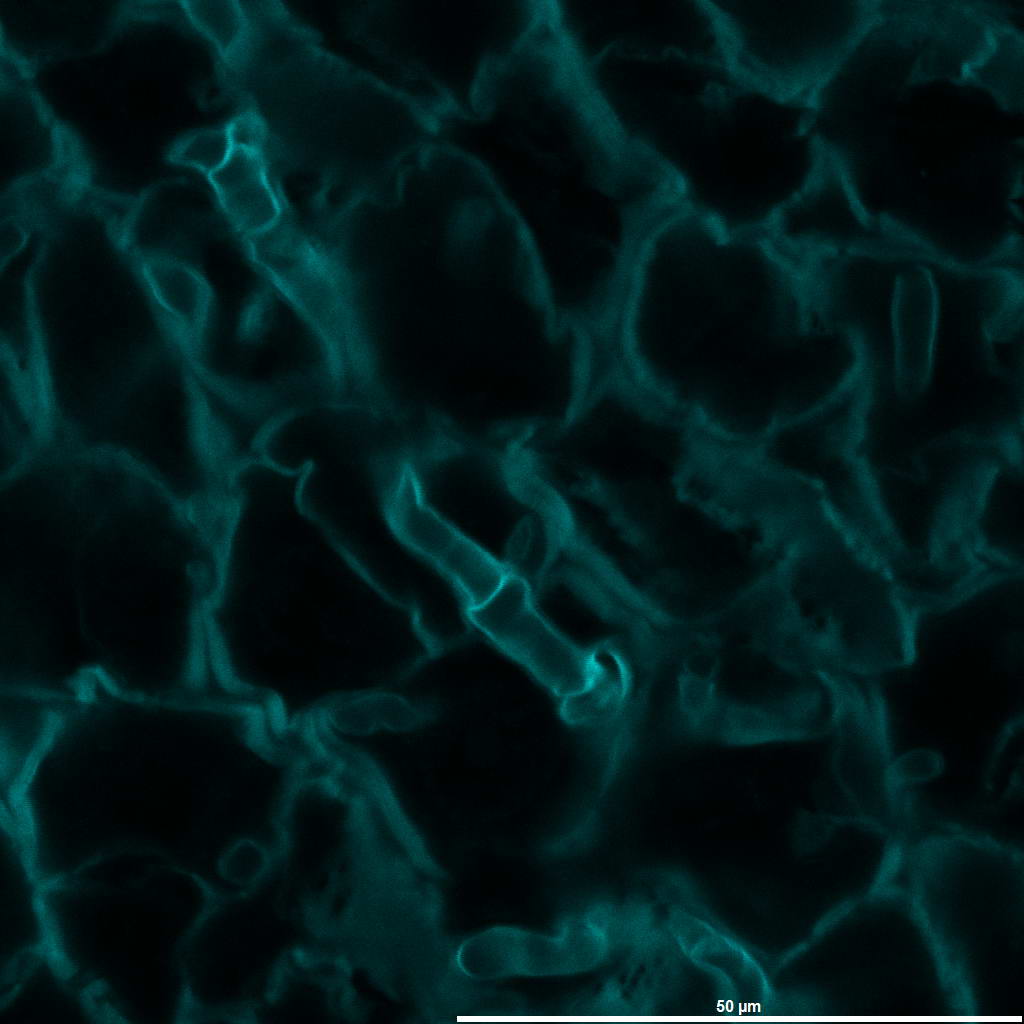

Supplement: Supplementary file 1 [file plants-14-01083-s001.zip › Appendix_A-CLSM-set-images_Fig8/Tfrezzi_peanut_CalcW_Ser09_z39.jpg]

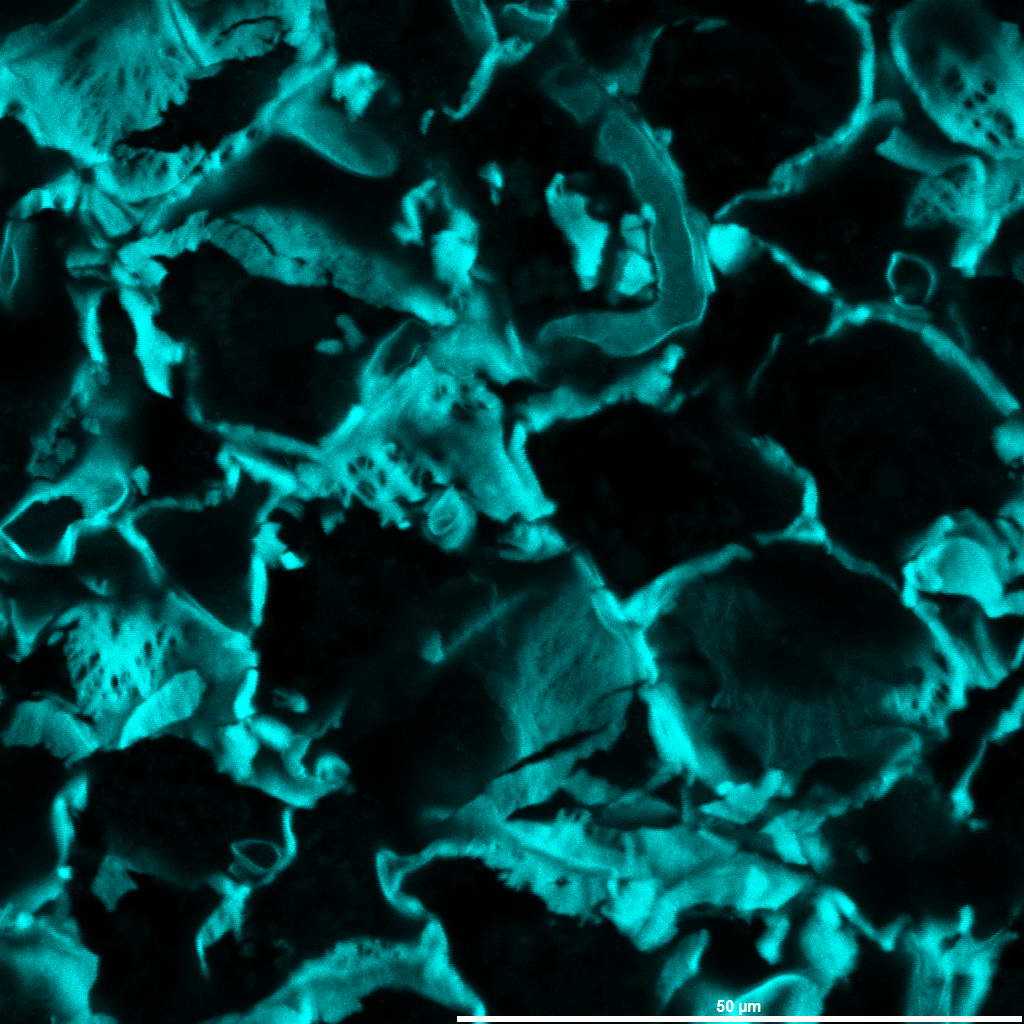

Supplement: Supplementary file 1 [file plants-14-01083-s001.zip › Appendix_A-CLSM-set-images_Fig8/Tfrezzi_peanut_CalcW_Ser09_z4.jpg]

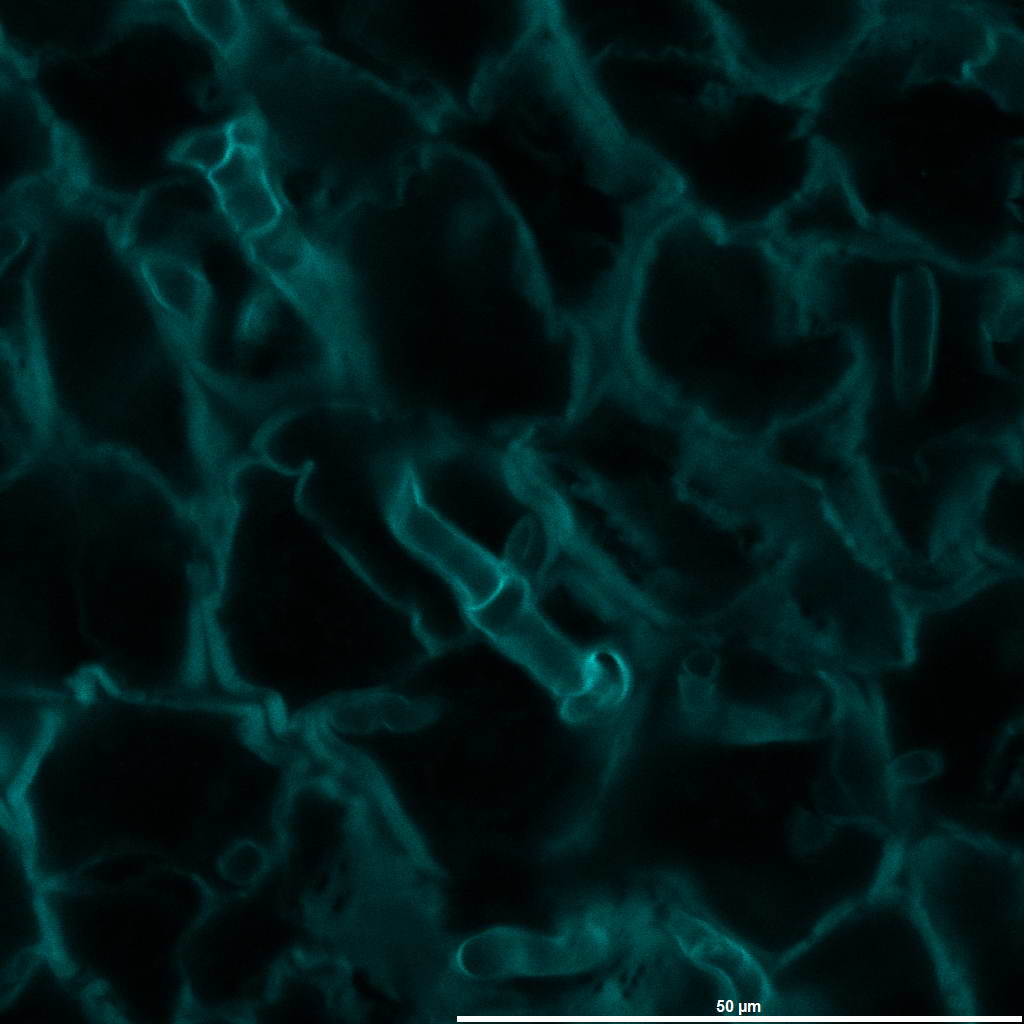

Supplement: Supplementary file 1 [file plants-14-01083-s001.zip › Appendix_A-CLSM-set-images_Fig8/Tfrezzi_peanut_CalcW_Ser09_z40.jpg]

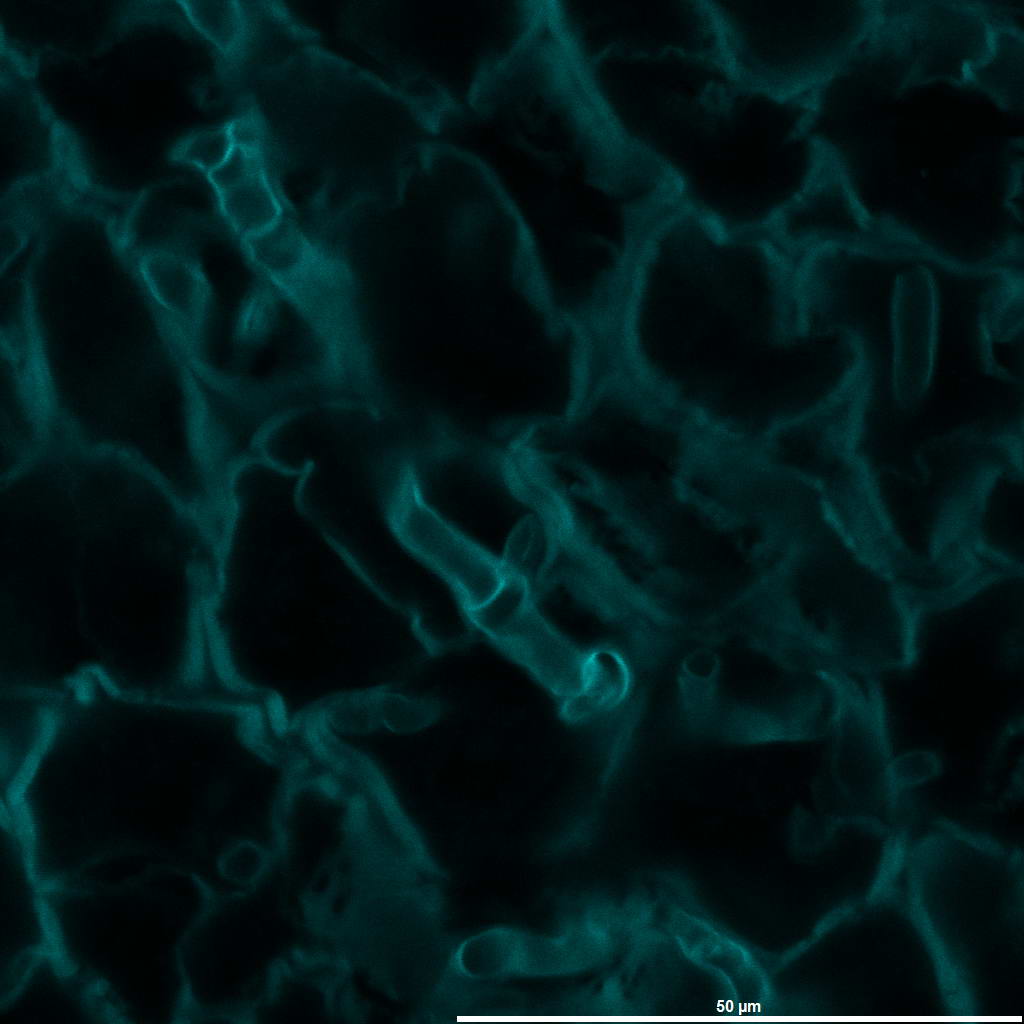

Supplement: Supplementary file 1 [file plants-14-01083-s001.zip › Appendix_A-CLSM-set-images_Fig8/Tfrezzi_peanut_CalcW_Ser09_z41.jpg]

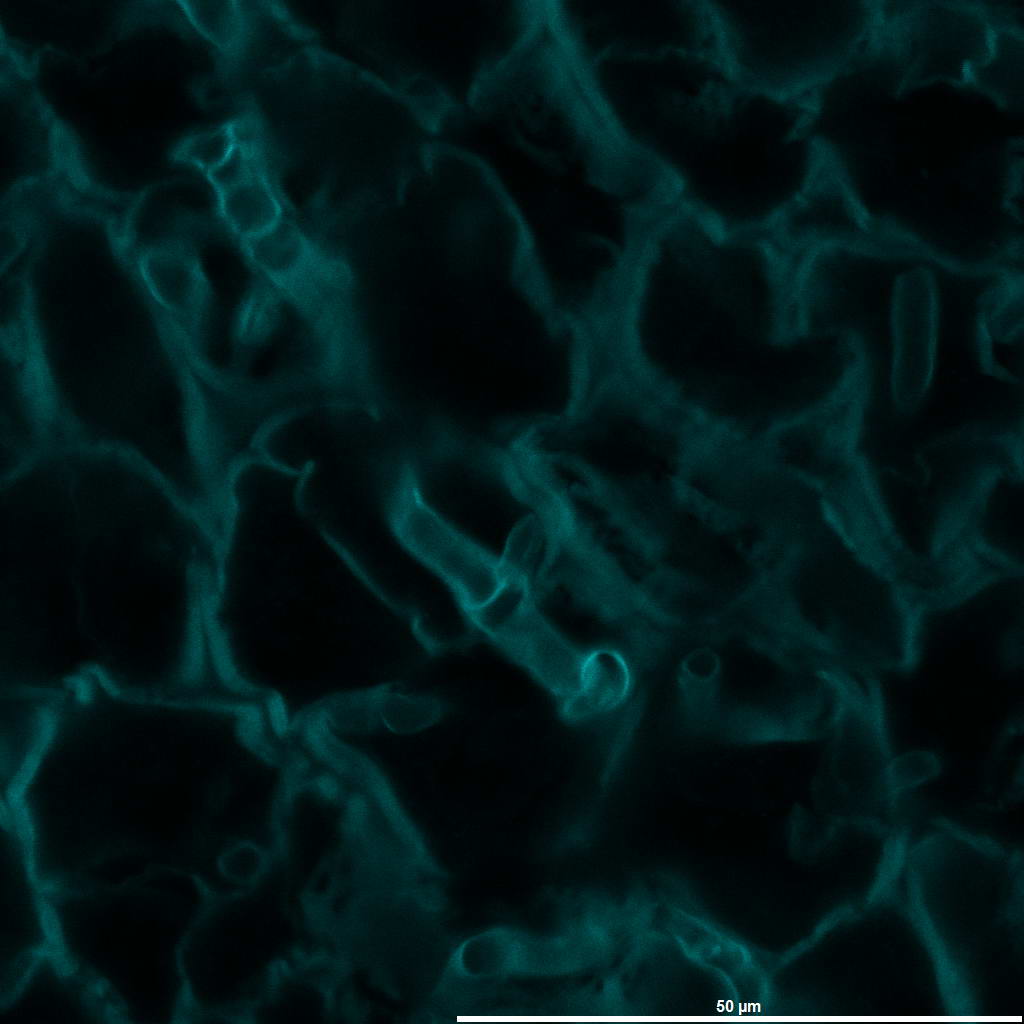

Supplement: Supplementary file 1 [file plants-14-01083-s001.zip › Appendix_A-CLSM-set-images_Fig8/Tfrezzi_peanut_CalcW_Ser09_z42.jpg]

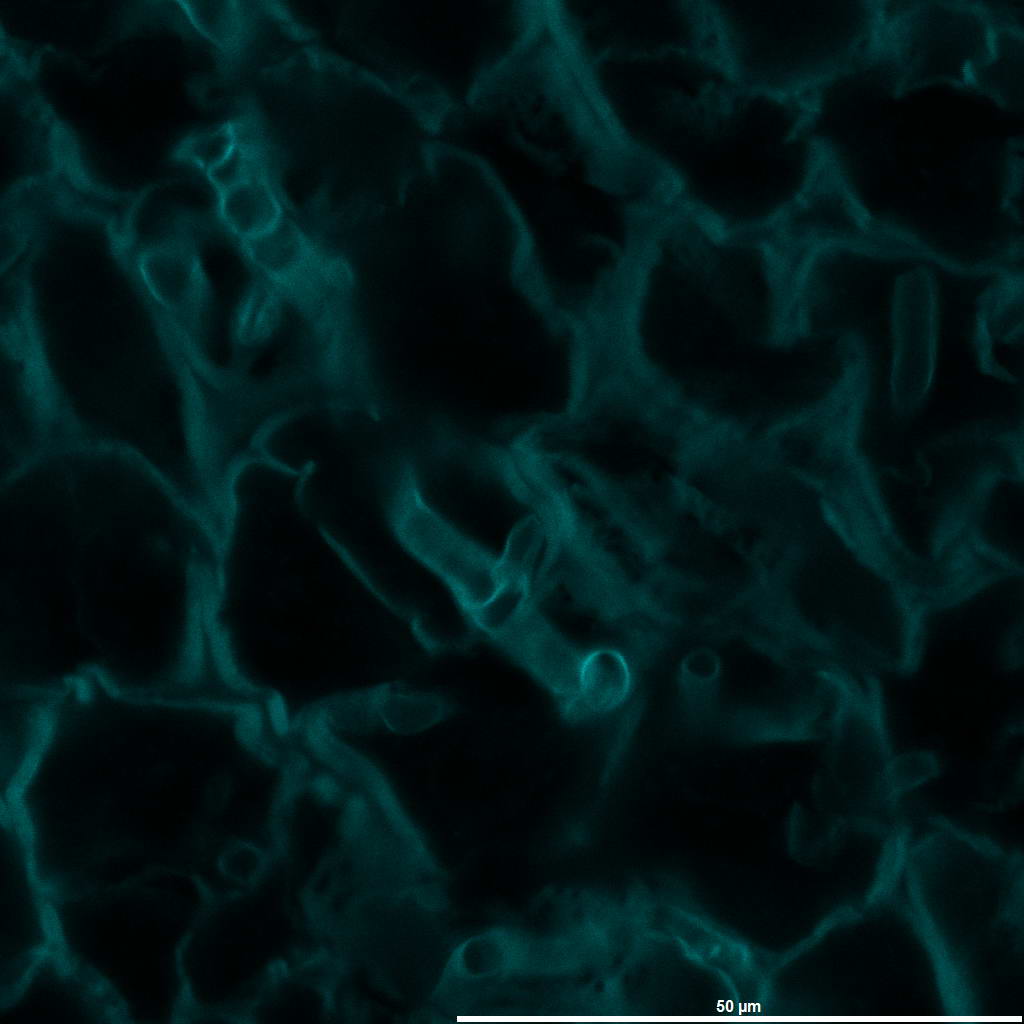

Supplement: Supplementary file 1 [file plants-14-01083-s001.zip › Appendix_A-CLSM-set-images_Fig8/Tfrezzi_peanut_CalcW_Ser09_z43.jpg]

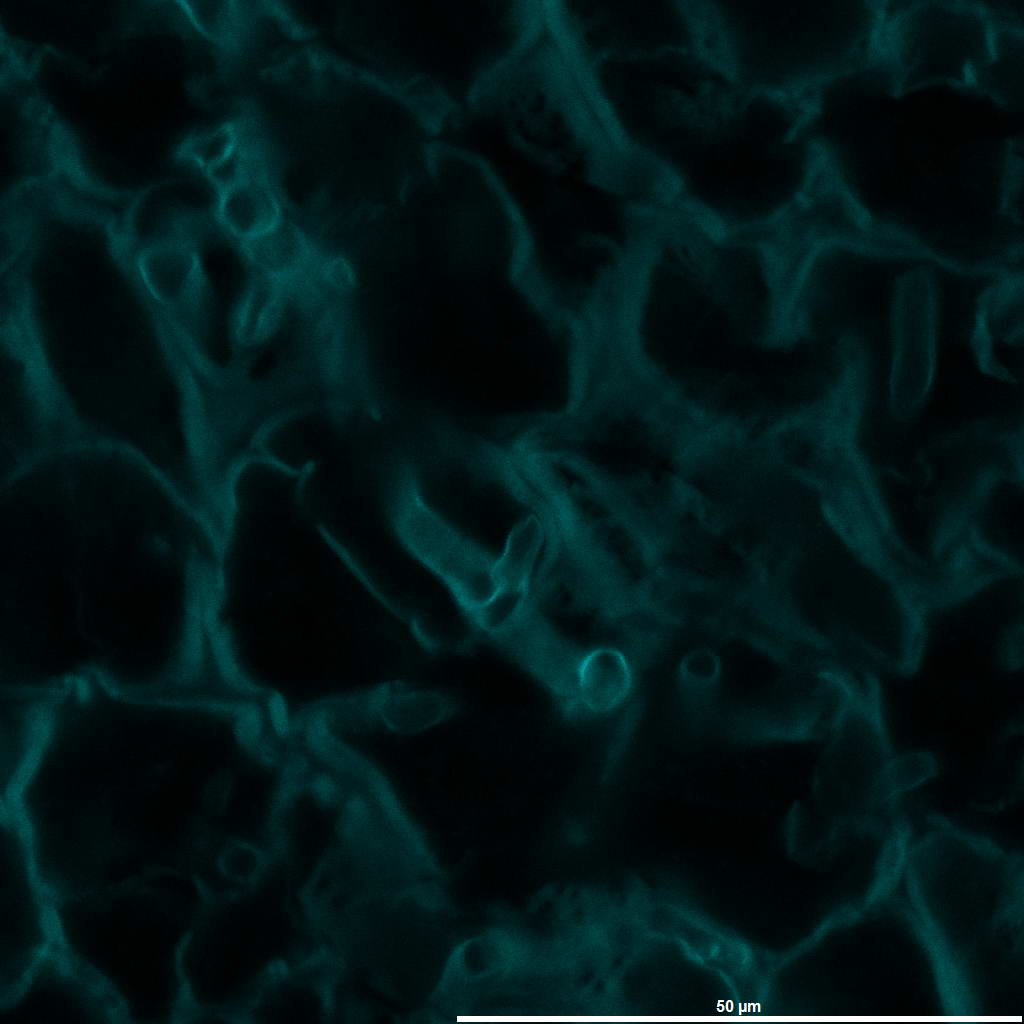

Supplement: Supplementary file 1 [file plants-14-01083-s001.zip › Appendix_A-CLSM-set-images_Fig8/Tfrezzi_peanut_CalcW_Ser09_z44.jpg]

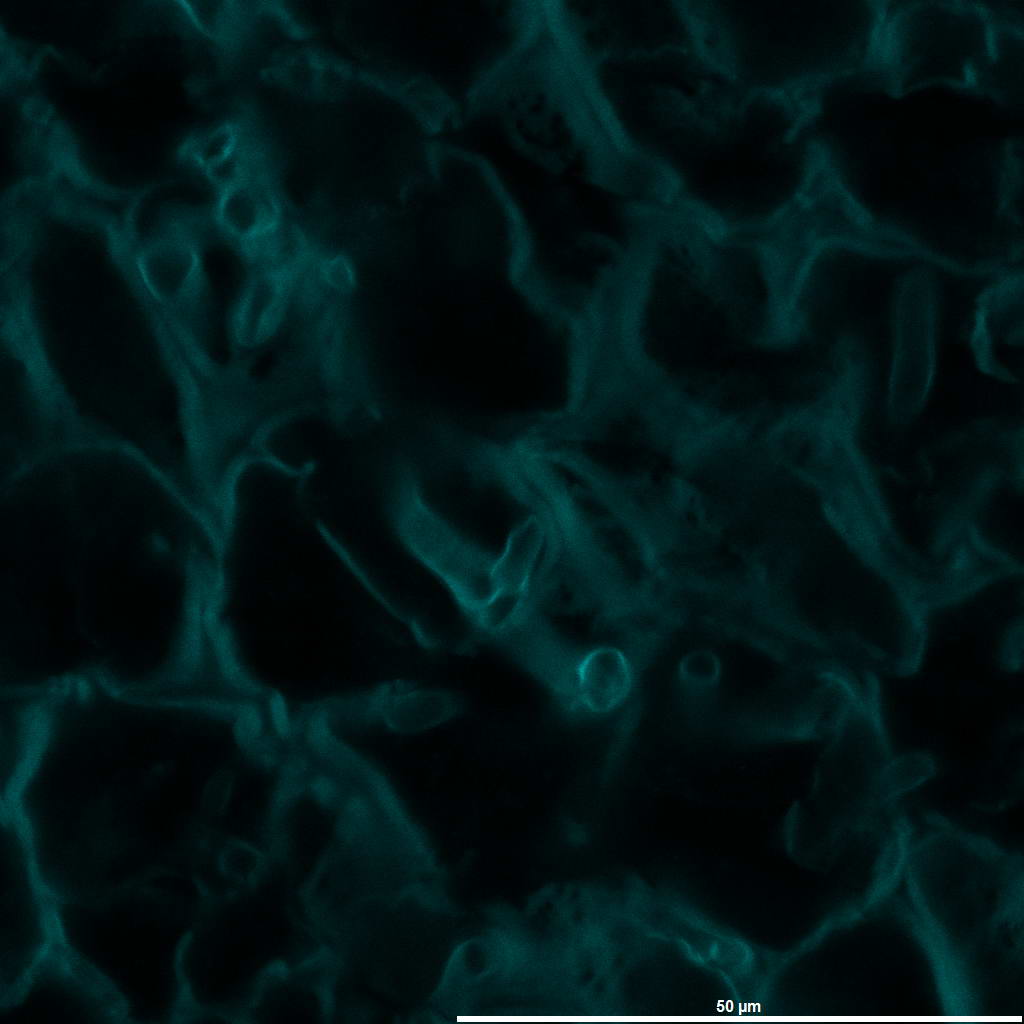

Supplement: Supplementary file 1 [file plants-14-01083-s001.zip › Appendix_A-CLSM-set-images_Fig8/Tfrezzi_peanut_CalcW_Ser09_z45.jpg]

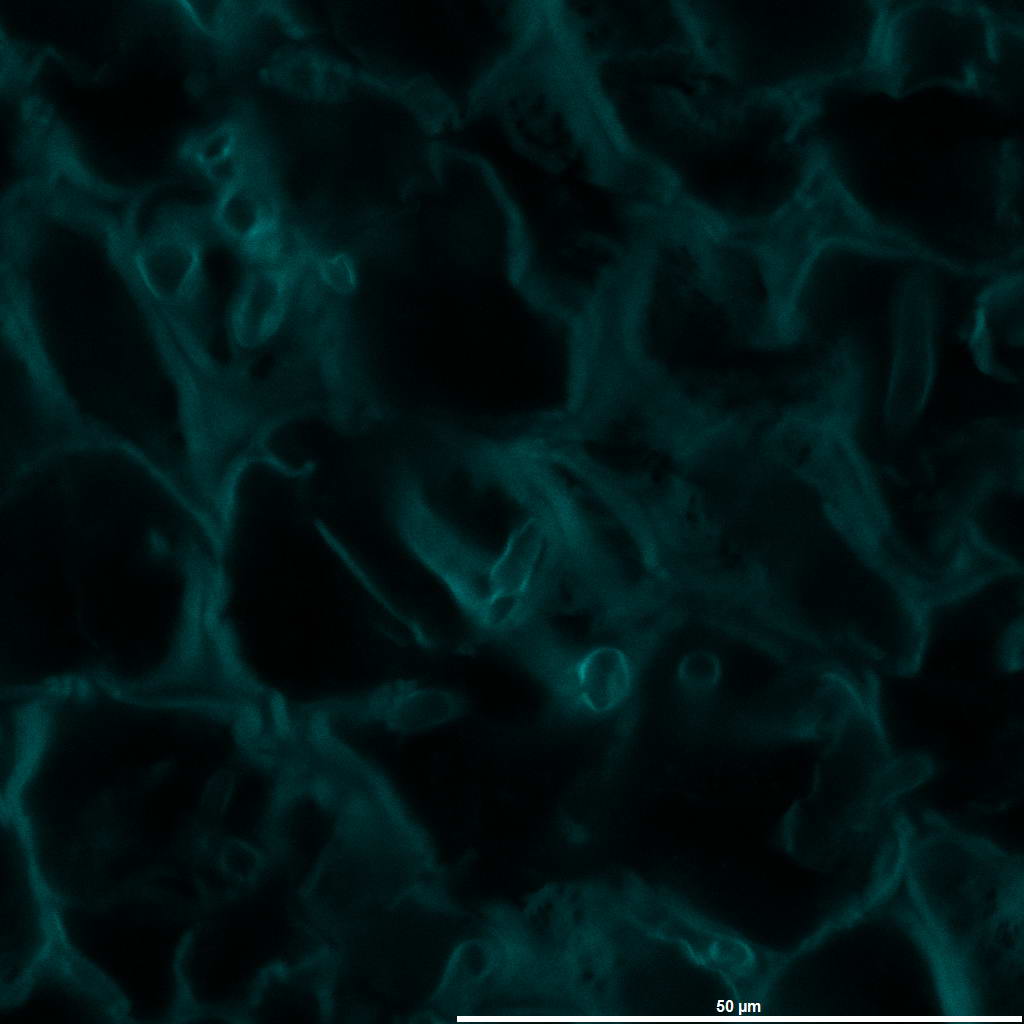

Supplement: Supplementary file 1 [file plants-14-01083-s001.zip › Appendix_A-CLSM-set-images_Fig8/Tfrezzi_peanut_CalcW_Ser09_z46.jpg]

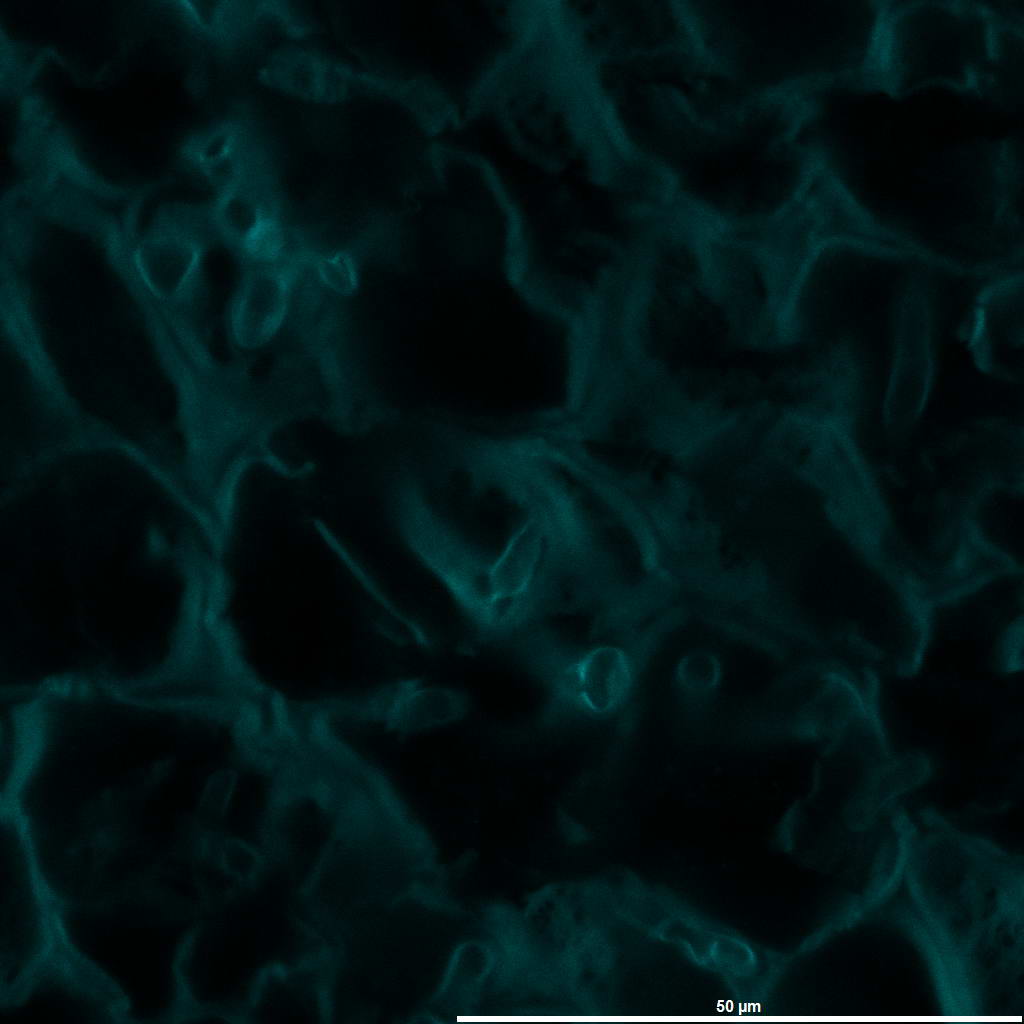

Supplement: Supplementary file 1 [file plants-14-01083-s001.zip › Appendix_A-CLSM-set-images_Fig8/Tfrezzi_peanut_CalcW_Ser09_z47.jpg]

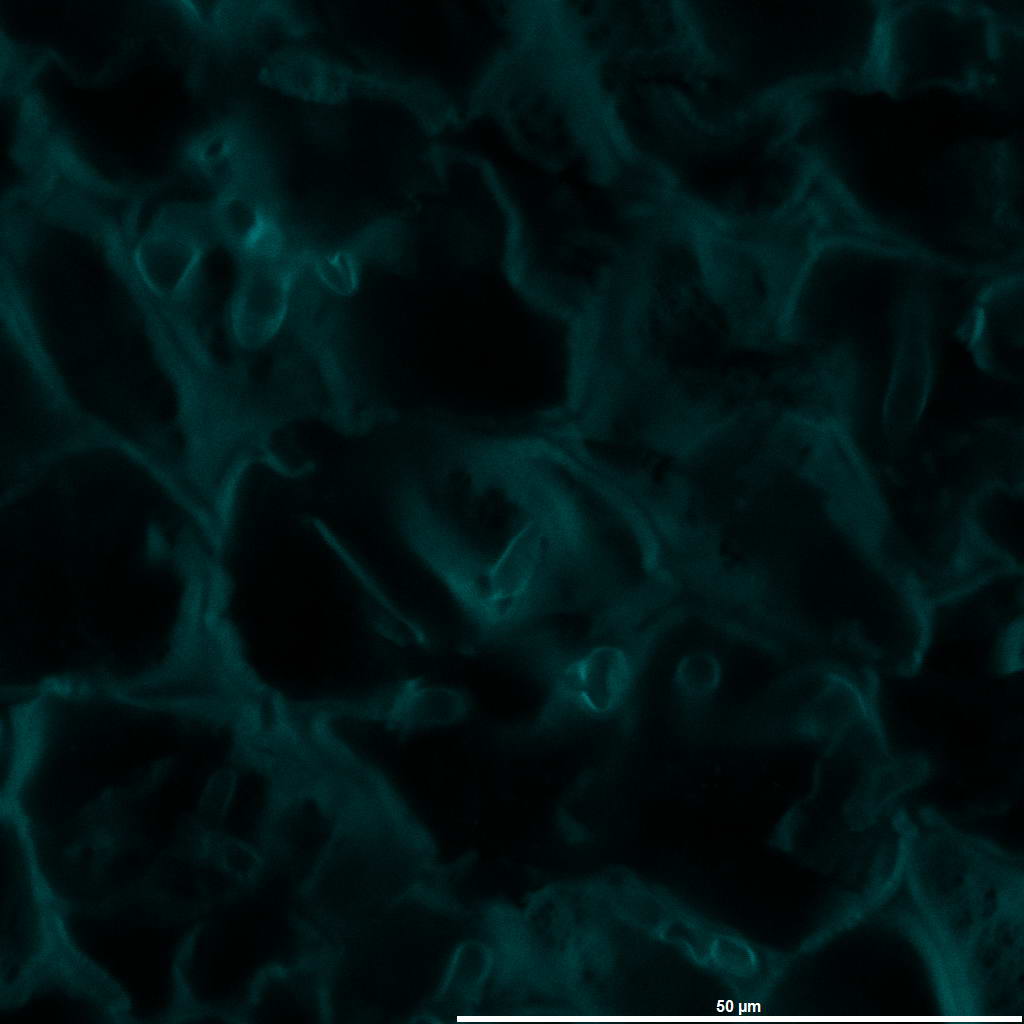

Supplement: Supplementary file 1 [file plants-14-01083-s001.zip › Appendix_A-CLSM-set-images_Fig8/Tfrezzi_peanut_CalcW_Ser09_z48.jpg]

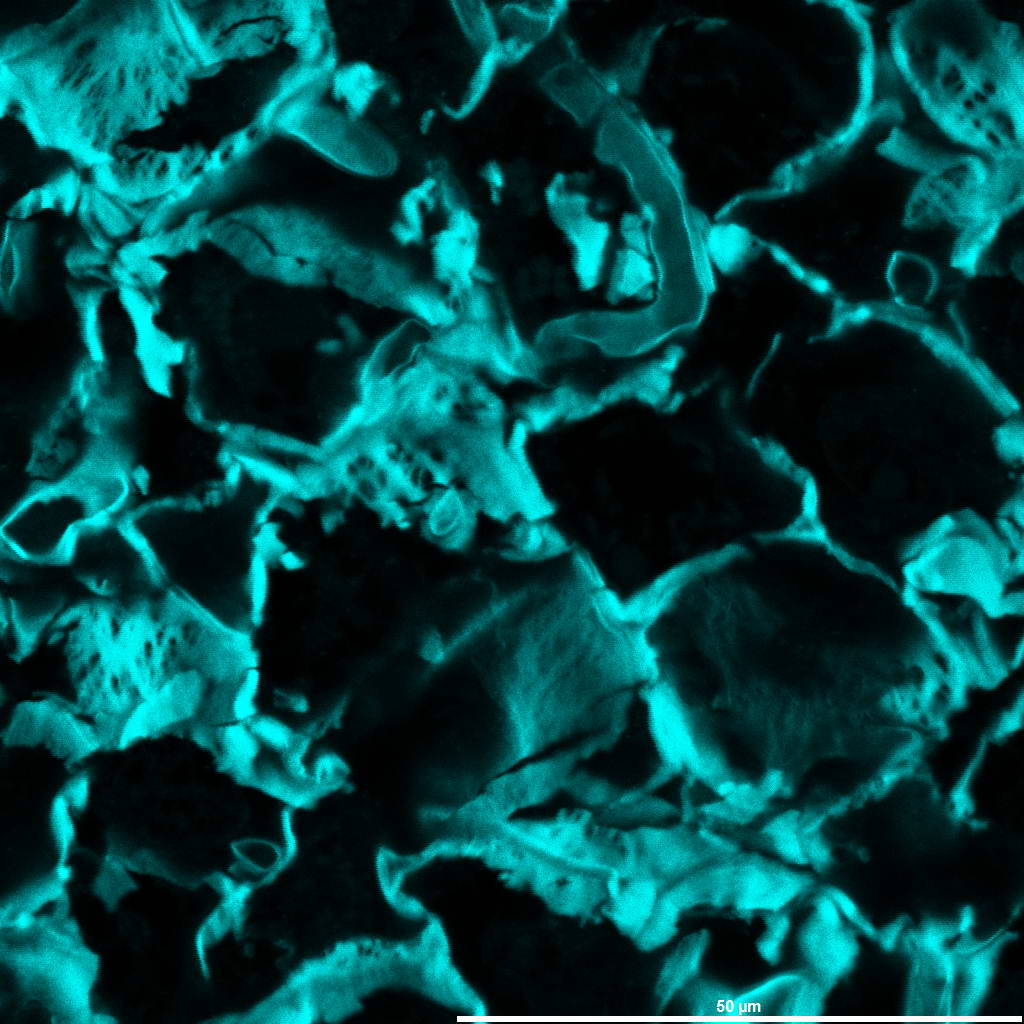

Supplement: Supplementary file 1 [file plants-14-01083-s001.zip › Appendix_A-CLSM-set-images_Fig8/Tfrezzi_peanut_CalcW_Ser09_z5.jpg]

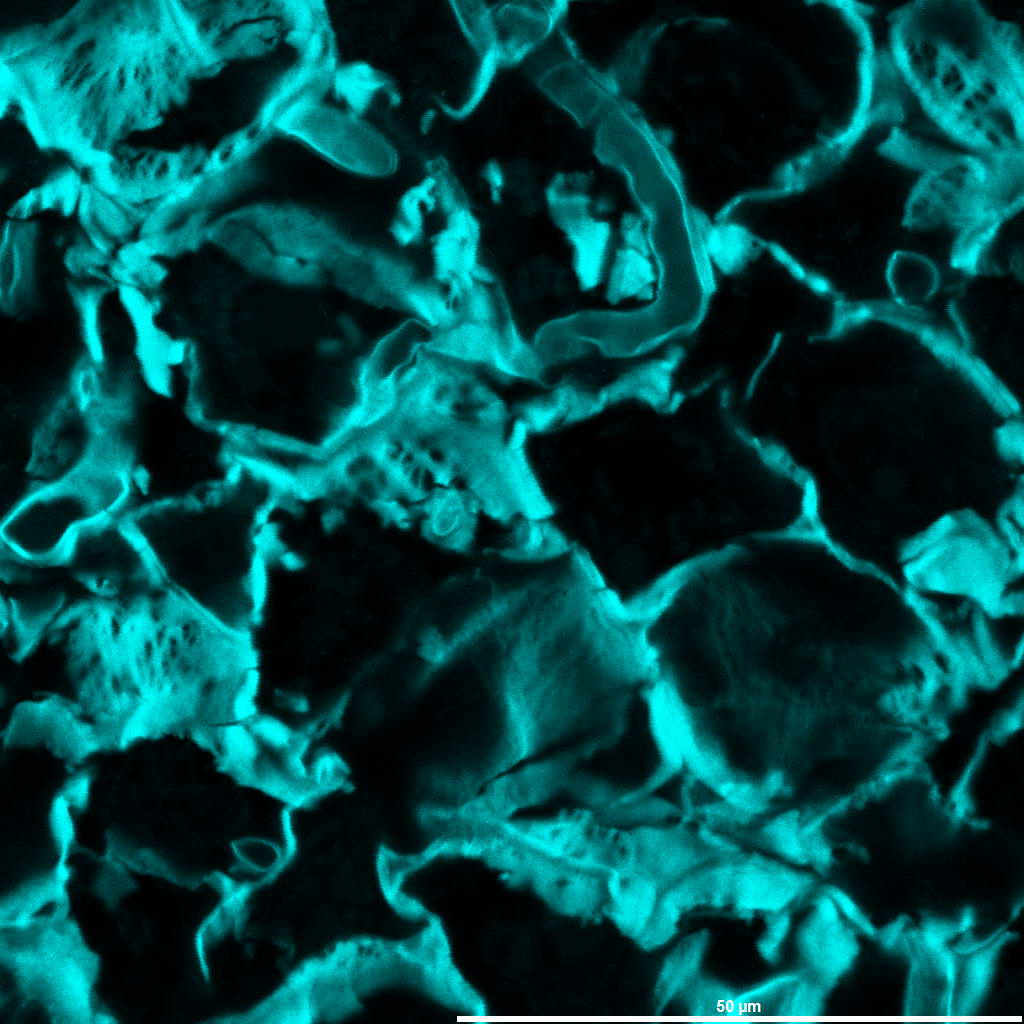

Supplement: Supplementary file 1 [file plants-14-01083-s001.zip › Appendix_A-CLSM-set-images_Fig8/Tfrezzi_peanut_CalcW_Ser09_z6.jpg]

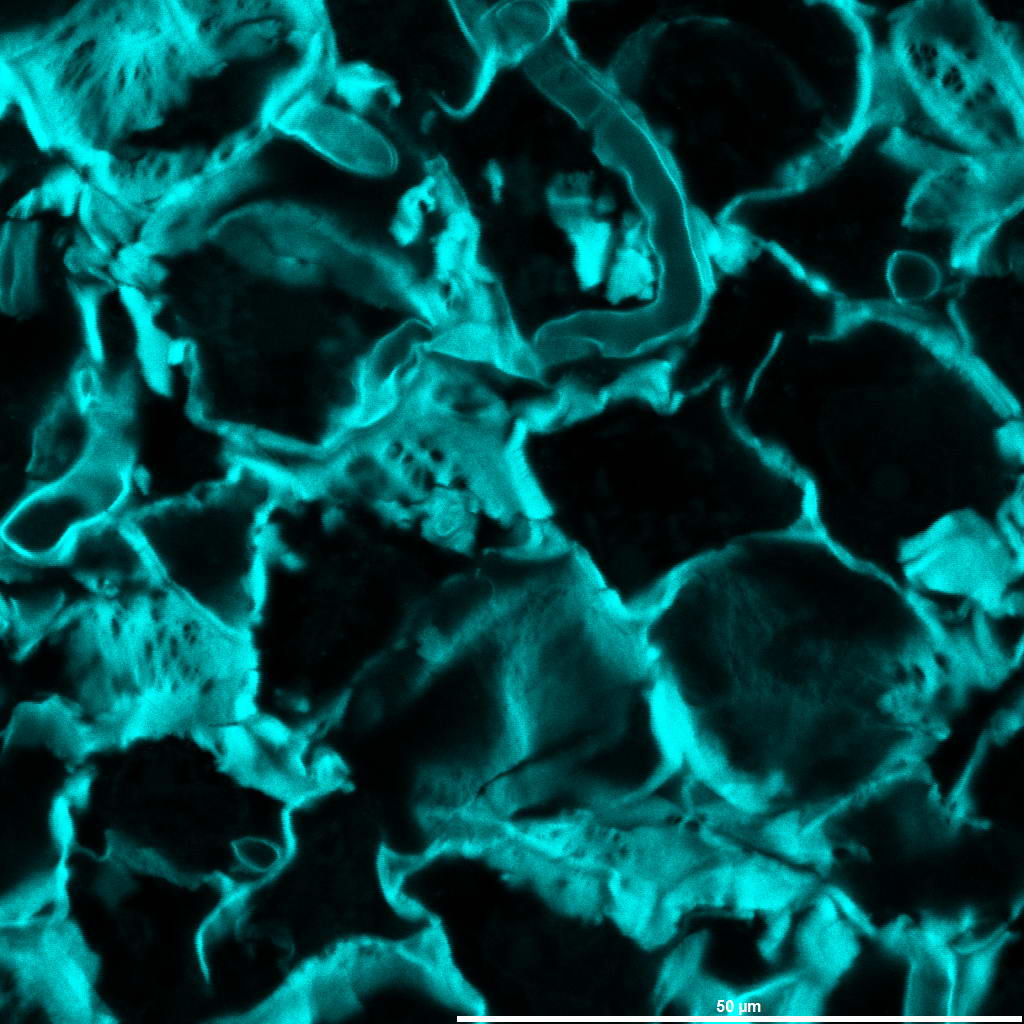

Supplement: Supplementary file 1 [file plants-14-01083-s001.zip › Appendix_A-CLSM-set-images_Fig8/Tfrezzi_peanut_CalcW_Ser09_z7.jpg]

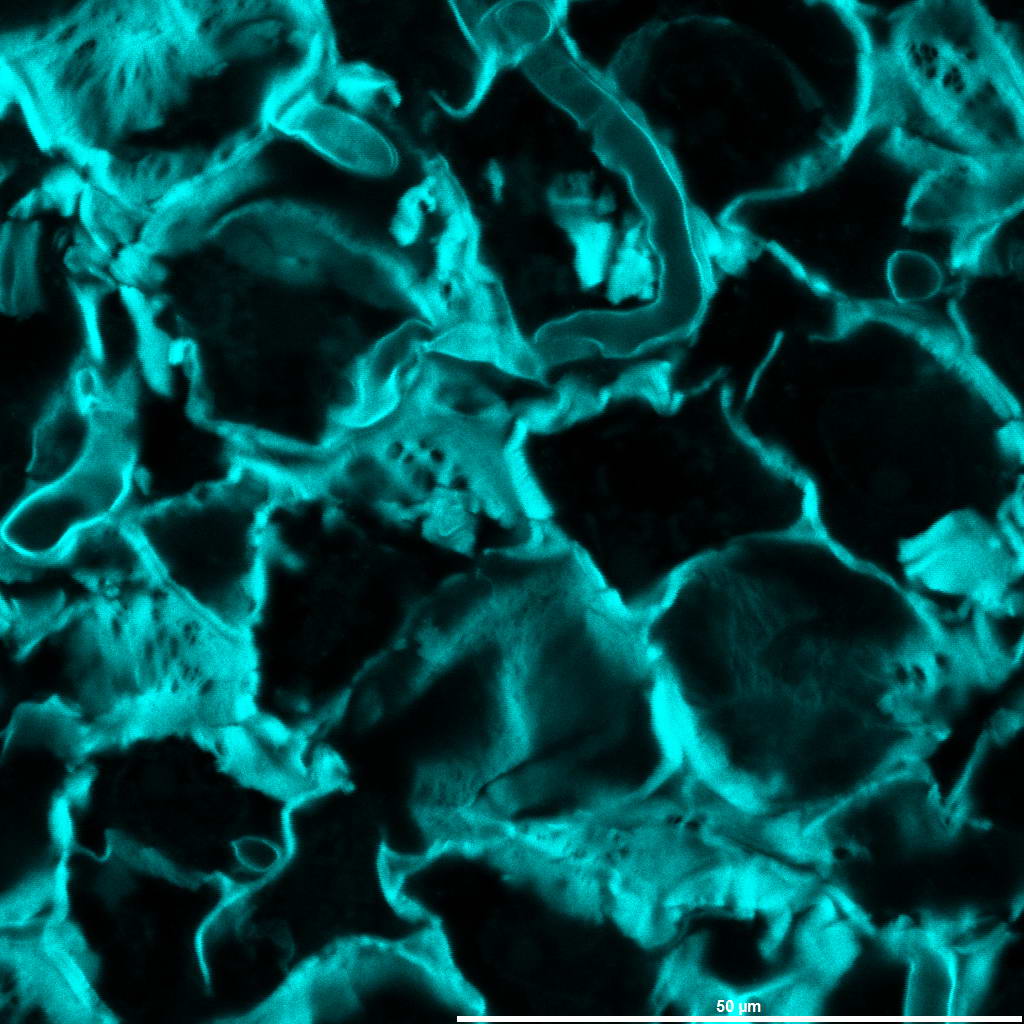

Supplement: Supplementary file 1 [file plants-14-01083-s001.zip › Appendix_A-CLSM-set-images_Fig8/Tfrezzi_peanut_CalcW_Ser09_z8.jpg]

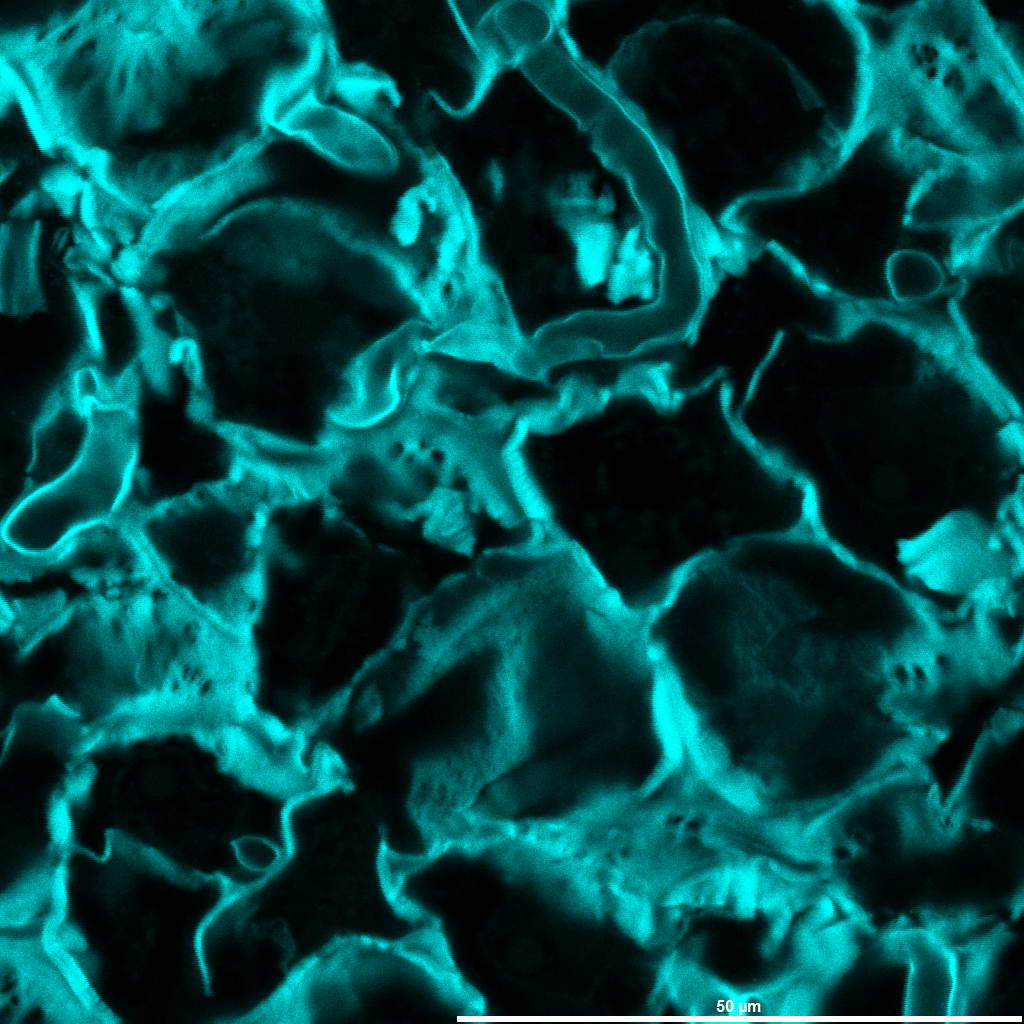

Supplement: Supplementary file 1 [file plants-14-01083-s001.zip › Appendix_A-CLSM-set-images_Fig8/Tfrezzi_peanut_CalcW_Ser09_z9.jpg]

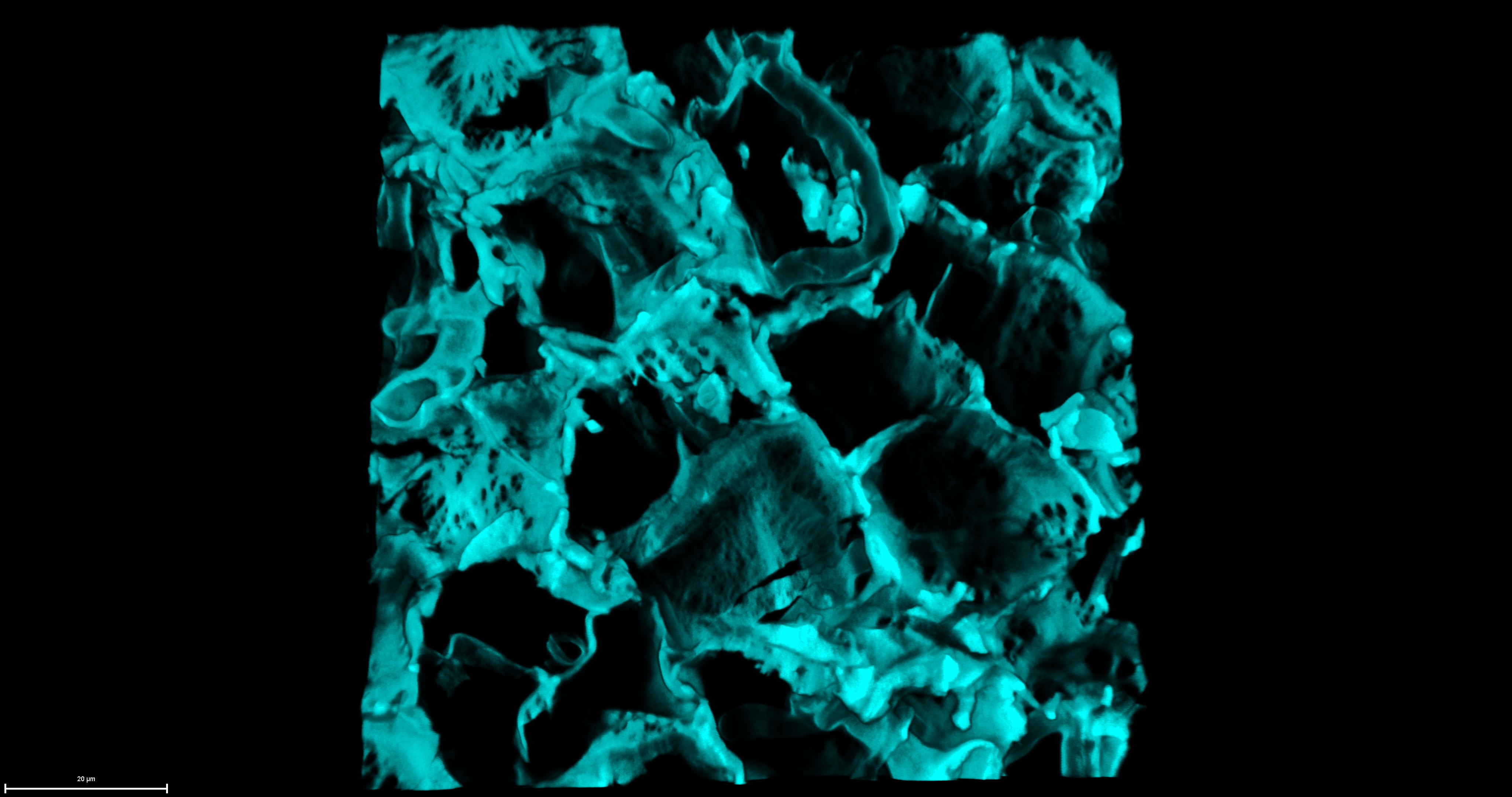

Supplement: Supplementary file 1 [file plants-14-01083-s001.zip › Appendix_B-CLSM-3Dimages_Fig8/Tfrezzi_peanut_CalcW_Ser09.jpg]

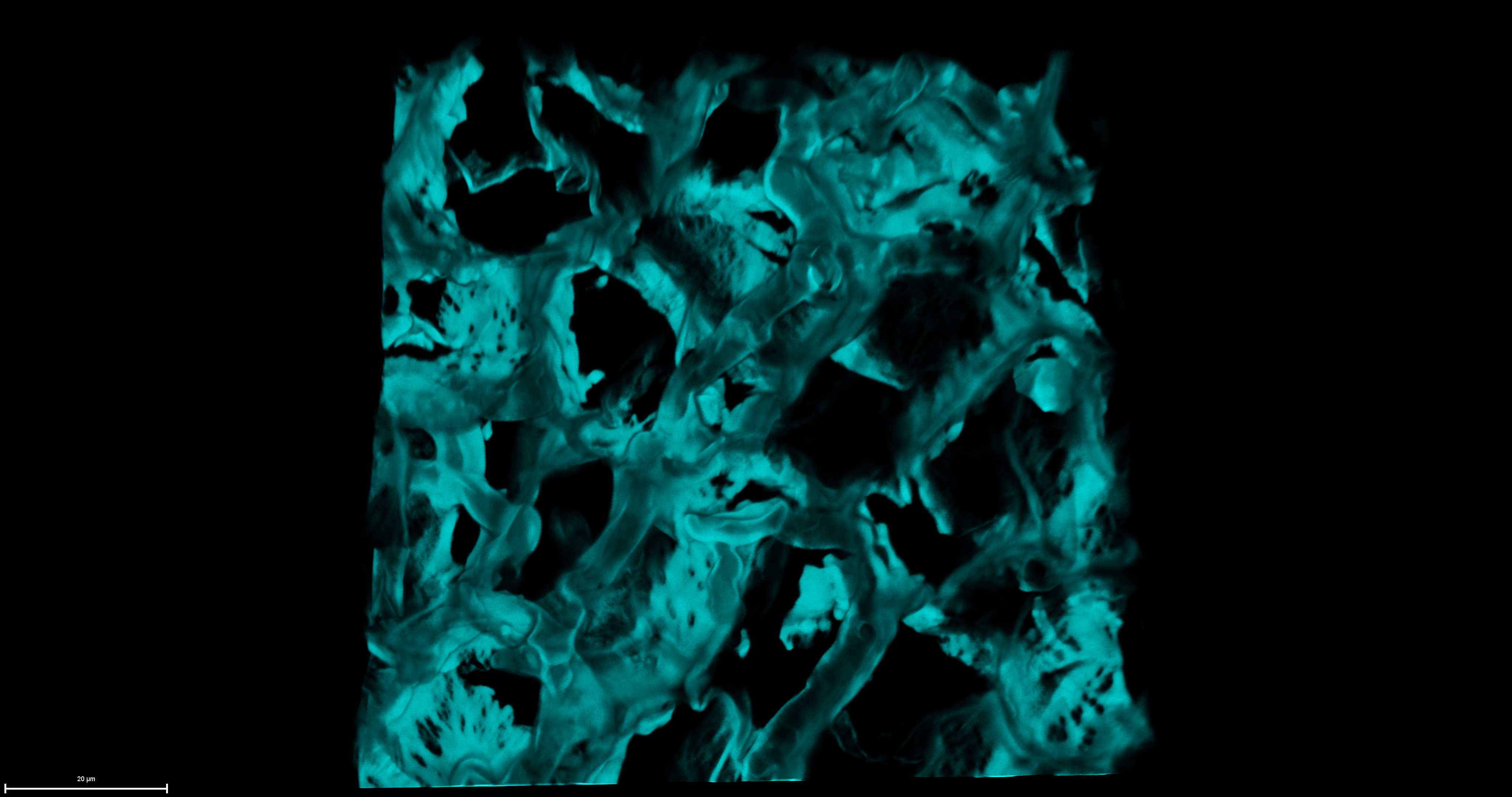

Supplement: Supplementary file 1 [file plants-14-01083-s001.zip › Appendix_B-CLSM-3Dimages_Fig8/Tfrezzi_peanut_CalcW_Ser09b.jpg]

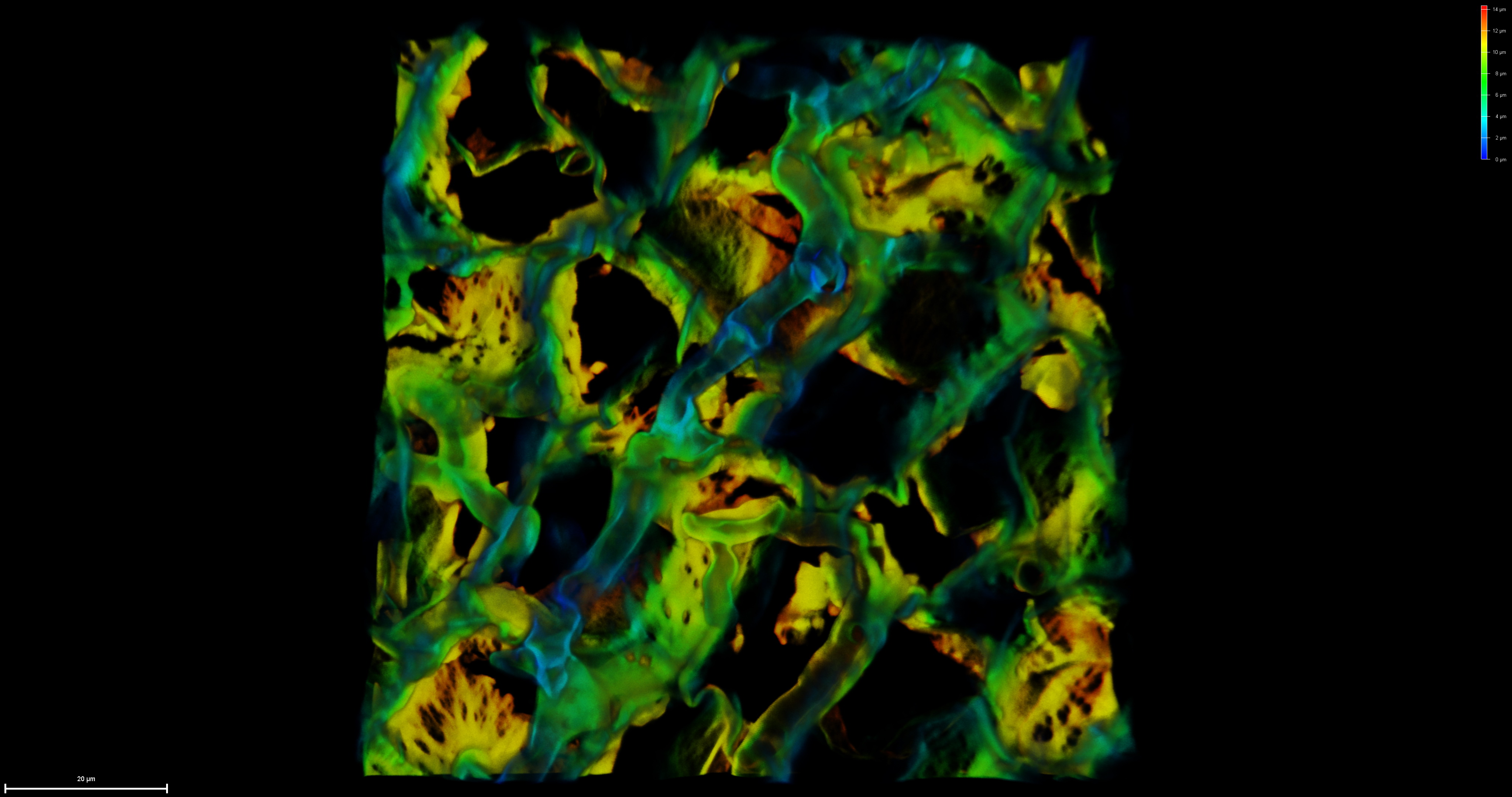

Supplement: Supplementary file 1 [file plants-14-01083-s001.zip › Appendix_B-CLSM-3Dimages_Fig8/Tfrezzi_peanut_CalcW_Ser09_DC.jpg]

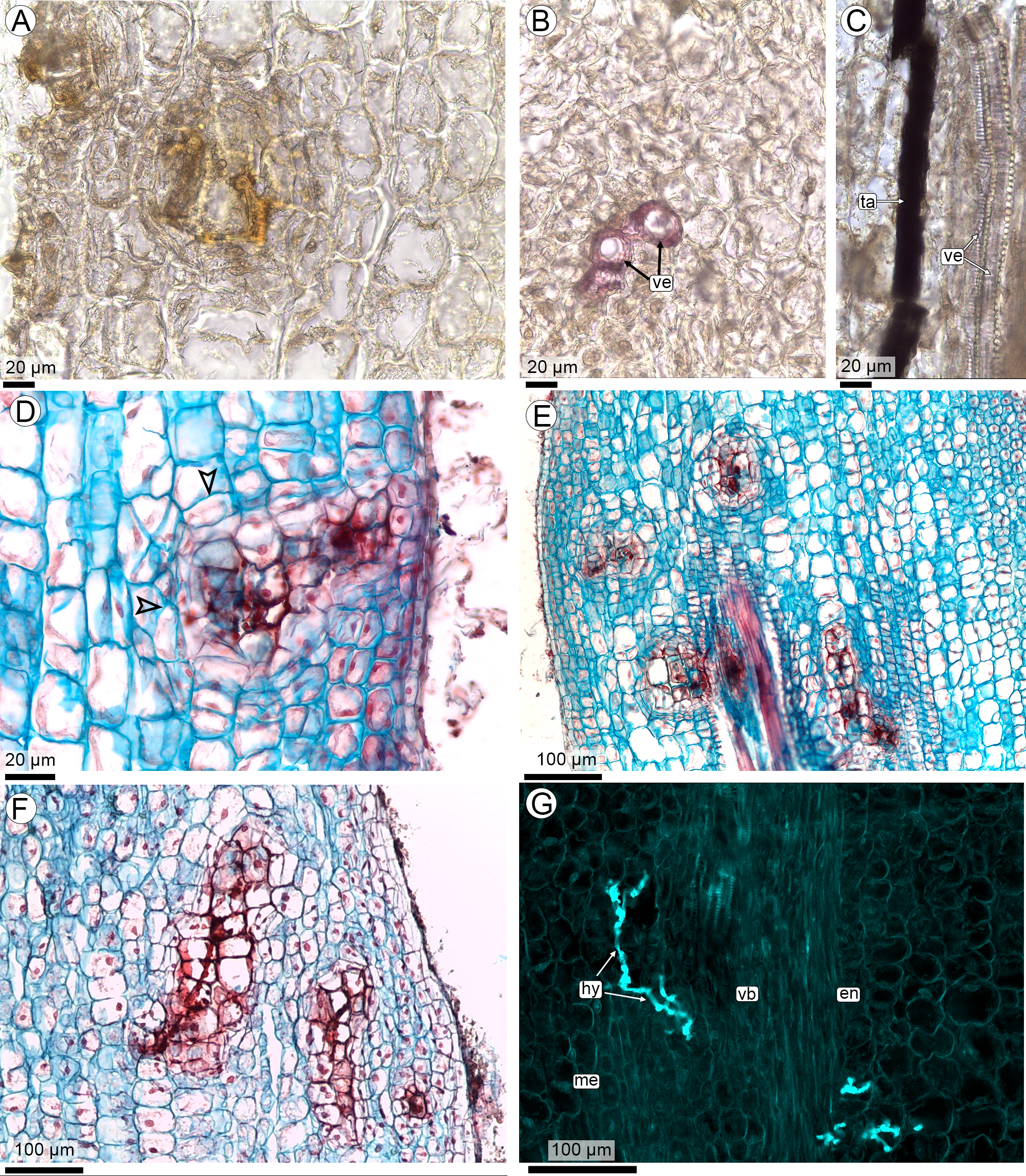

Supplement: Supplementary file 1 [file plants-14-01083-s001.zip › Rev-Figure S1.jpg]

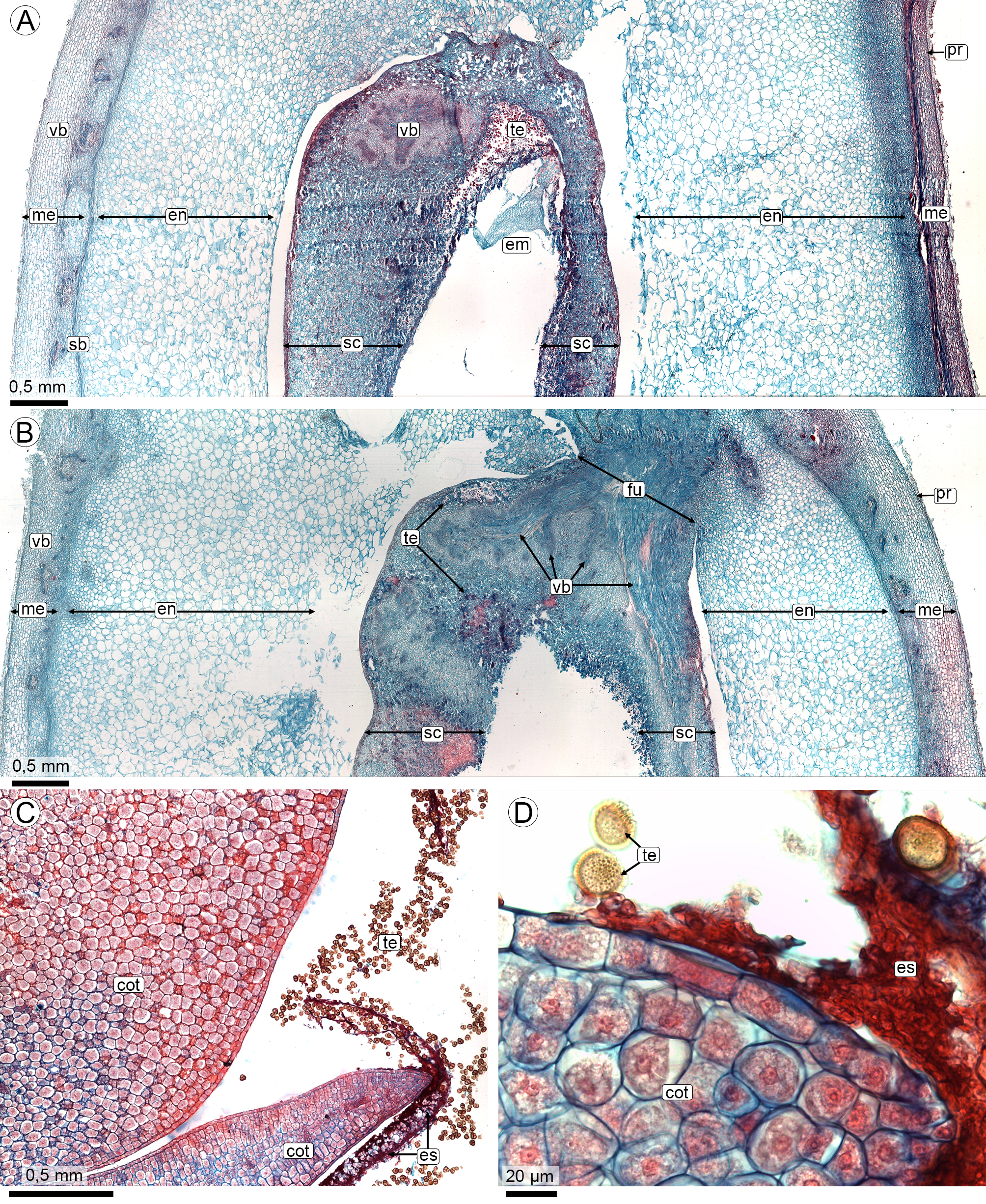

Supplement: Supplementary file 1 [file plants-14-01083-s001.zip › Rev-Figure S2.jpg]
